# ERG responses to high-frequency flickers require FAT3 signaling in mouse retinal bipolar cells

**DOI:** 10.1085/jgp.202413642

**Published:** 2025-02-04

**Authors:** Evelyn C. Avilés, Sean K. Wang, Sarina Patel, Sebastian Cordero, Shuxiang Shi, Lucas Lin, Vladimir J. Kefalov, Lisa V. Goodrich, Constance L. Cepko, Yunlu Xue

**Affiliations:** 1 Department of Neurobiology, Blavatnik Institute, Harvard Medical School, Boston, MA, USA; 2 Facultad de Ciencias Biológicas, Pontificia Universidad Católica de Chile, Santiago, Chile; 3 Departments of Genetics and Ophthalmology, Harvard Medical School, Boston, MA, USA; 4 Howard Hughes Medical Institute, Boston, MA, USA; 5 Lingang Laboratory, Shanghai, China; 6 School of Life Science and Technology, ShanghaiTech University, Shanghai, China; 7 Gavin Herbert Eye Institute and Center for Translational Vision Research, University of California, Irvine, Irvine, CA, USA

## Abstract

Vision is initiated by the reception of light by photoreceptors and subsequent processing via downstream retinal neurons. Proper circuit organization depends on the multifunctional tissue polarity protein FAT3, which is required for amacrine cell connectivity and retinal lamination. Here, we investigated the retinal function of *Fat3* mutant mice and found decreases in both electroretinography and perceptual responses to high-frequency flashes. These defects did not correlate with abnormal amacrine cell wiring, pointing instead to a role in bipolar cell subtypes that also express FAT3. The role of FAT3 in the response to high temporal frequency flashes depends upon its ability to transduce an intracellular signal. Mechanistically, FAT3 binds to the synaptic protein PTPσ intracellularly and is required to localize GRIK1 to OFF-cone bipolar cell synapses with cone photoreceptors. These findings expand the repertoire of FAT3’s functions and reveal its importance in bipolar cells for high-frequency light response.

## Teaser

This study reports that the loss of FAT3, a multifunctional tissue polarity protein, acts via its intracellular domain and retinal bipolar cells to affect mouse electroretinography (ERG) responses and visual perception of high-frequency flickers.

## Introduction

**Figure 1.**
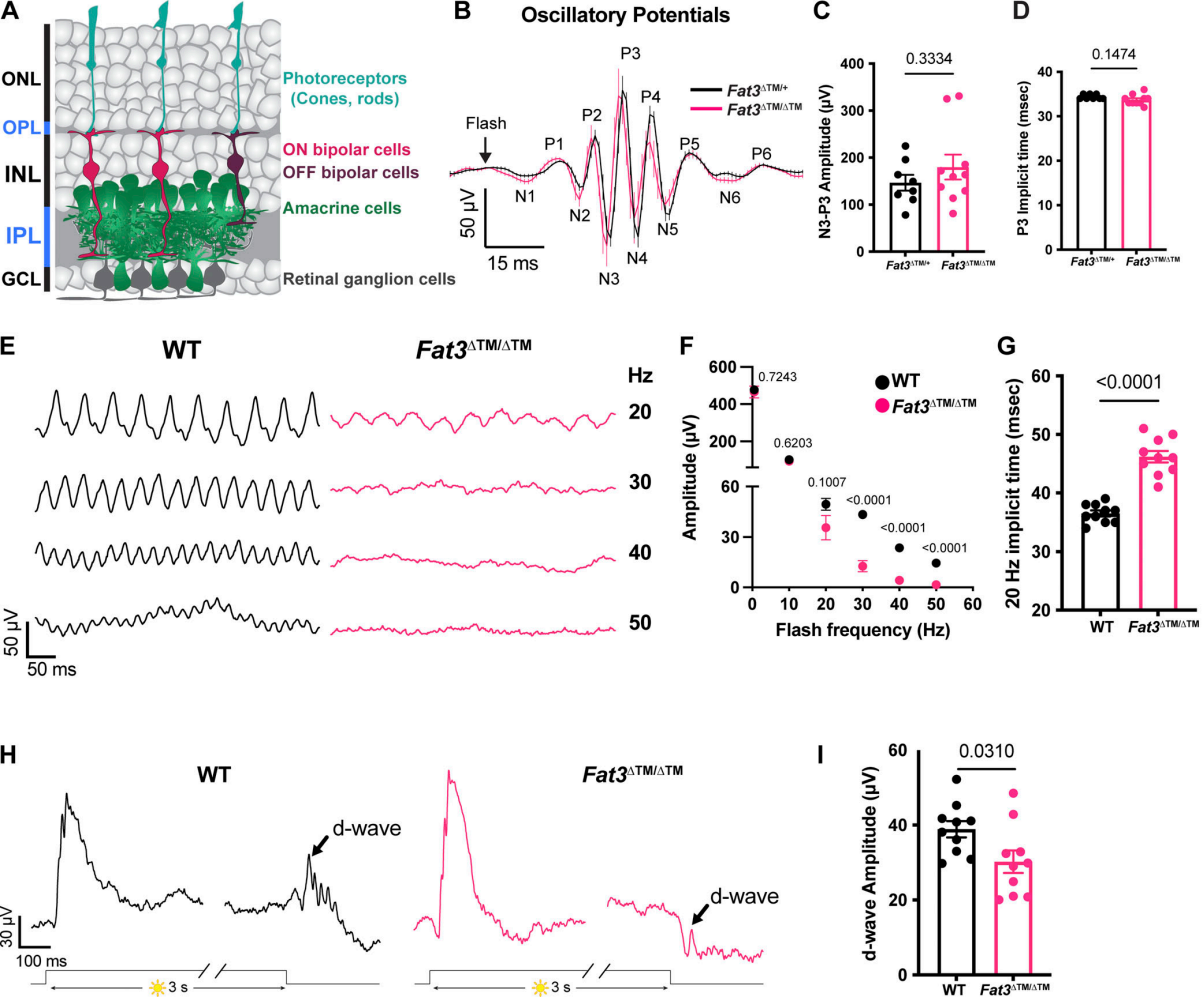
**Flicker ERG and vision at high frequency and step ERG of *Fat3*-deficient mice. (A)** Schematic representation of retinal neurons and their layers. **(B)** Averaged oscillatory potentials of *Fat3*^∆TM/+^ (*n* = 8) and *Fat3*^∆TM/∆TM^ (*n* = 10) eyes at scotopic condition are elicited by 0.1 cd s/m^2^ flashes (same as Fig. S1 B). Line: Mean values. Error bar: SEM. **(C)** Peak amplitude (N3-P3) of oscillatory potentials (same as Fig. 1 B, 0.1 cd s/m^2^ scotopic condition). *Fat3*^∆TM/+^: 146.7 ± 16.8 µV, *n* = 8 eyes; *Fat3*^∆TM/∆TM^: 179.9 ± 26.5 µV, *n* = 10 eyes. Unpaired two-tailed Student’s *t* test. Error bar: SEM. The numerical values above the bar graph for this and the rest of figures: P values. **(D)** Peak implicit time (P3) of oscillatory potentials. *Fat3*^∆TM/+^: 34.38 ± 0.18 ms, *n* = 8 eyes; *Fat3*^∆TM/∆TM^: 33.70 ± 0.37 µV, *n* = 10 eyes. Unpaired two-tailed Student’s *t* test. Error bar: SEM. **(E)** Representative flicker ERG raw traces of WT control and *Fat3*^∆TM/∆TM^ eyes elicited by 3.162 cd s/m^2^ flashes at 20, 30, 40, and 50 Hz frequencies. **(F)** Flicker ERG amplitude at 0.5, 10, 20, 30, 40, and 50 Hz for WT control (*n* = 10) and *Fat3*^∆TM/∆TM^ (*n* = 10) eyes. Unpaired two-tailed Student’s *t* test. Error bar: SEM. **(G)** Flicker ERG implicit time (first peak) at 20 Hz for WT control (36.5 ± 0.5 ms, *n* = 10) and *Fat3*^∆TM/∆TM^ (46.2 ± 1.0 ms, *n* = 10) eyes. Unpaired two-tailed Student’s *t* test. Error bar: SEM. **(H)** Representative step ERG raw traces of WT (*n* = 10) control and *Fat3*^∆TM/∆TM^ (*n* = 10) eyes elicited by a 3-s step light at 1,000 cd/m^2^ intensity. **(I)** Quantification of step ERG d-wave amplitudes of WT (38.9 ± 2.2 µV, *n* = 10) control and *Fat3*^∆TM/∆TM^ (30.2 ± 3.0 µV, *n* = 10) eyes elicited by a 3-s step of light at 1,000 cd/m^2^ intensity. Unpaired two-tailed Student’s *t* test. Error bar: SEM.

The remarkable ability to detect light over a wide range of intensities and frequencies is accomplished by highly specialized cell types within the retina. Retinal circuits encode signals originating in the photoreceptors, which synapse with bipolar cells (BCs). BCs are classified as ON or OFF depending upon their response to photoreceptor signals, thereby setting the stage for downstream processing events. Critical to these transformations are the >80 types of retinal interneurons (Shekhar and Sanes, 2021; Shekhar et al., 2016; Yan et al., 2020; Tran et al., 2019), which are organized into laminae with the cell bodies of the horizontal, BCs, and amacrine cells (ACs) located in the inner nuclear layer (INL). In the adjacent outer nuclear layer (ONL) reside the cell bodies of the photoreceptors. Retinal interneurons form synaptic connections in two layers: in the outer plexiform layer (OPL) among BCs, horizontal cells, and photoreceptors, and in the inner plexiform layer (IPL) among BCs, ACs, and the output neurons of the retina, the retinal ganglion cells (RGCs) (Fig. 1 A). Despite the stereotyped and conserved organization of retinal neurons and their connections, it is unclear how important lamination is for the sense of vision. It is also unclear how the overall organization of retinal circuitry collectively enables specific visual responses, such as the detection and transmission of information about light stimuli that flicker at a high frequency (Demb and Singer, 2015).

Several features of retinal organization depend upon FAT3, a tissue polarity protein (Avilés et al., 2022; Deans et al., 2011; Krol et al., 2016). FAT cadherins are transmembrane receptors that can sense cell position in the environment via their huge extracellular domains. They induce polarized changes in cell morphology during development via their intracellular domains (ICD), thereby creating cellular asymmetries that are aligned across the tissue. In *Fat3* mutant retinas, many AC somas migrate to ectopic locations in the IPL and the ganglion cell layer (GCL). They also fail to retract their trailing neurites, which go on to form ectopic synapses in two misplaced plexiform layers, one in the INL (the outer misplaced plexiform layer, OMPL) and one below the GCL (the inner misplaced plexiform layer, IMPL) (Avilés et al., 2022; Deans et al., 2011; Krol et al., 2016). These ectopic layers contain synapses between ACs and between ACs and rod BCs, which do not express FAT3 (Shekhar et al., 2016), but seem to “follow” their AC partners to abnormal locations (Deans et al., 2011). FAT3, which is localized to AC dendrites in the IPL, mediates these effects by localizing cytoskeletal effectors needed for migration and retraction (Avilés et al., 2022). The FAT3 intracellular domain also binds a variety of synaptic proteins (Avilés et al., 2022) and is therefore poised to control synapse composition and function independent of its effects on cell morphology. A direct role at the synapse has not been described, and it has not been clear how the loss of FAT3 impacts vision.

Here, we used retinal physiology and behavioral analyses to investigate the effects of FAT3 disruptions on retinal function and hence vision. Using *in vivo* electroretinography (ERG), we discovered an additional function for FAT3 at the cone-BC synapse that impacts retinal responses to high temporal frequency signals. Basic light responses were preserved in *Fat3* mutant mice despite the highly abnormal pattern of retinal lamination. However, overall retinal responses to 30-Hz flashes were severely reduced in amplitude, and many *Fat3* mutants behaved as if they saw constant illumination. Analysis of a series of conditional *Fat3* knock-out mice revealed that this phenotype is not due to altered AC position or connectivity. Instead, our evidence suggests that the OFF-cone pathway is abnormal, as revealed by an *in vivo* electroretinogram protocol that we developed. Further, we show that the FAT3 intracellular domain binds to the synaptic protein PTPσ and that in *Fat3* mutants, both PTPσ and the glutamate receptor subunit GRIK1 are present at reduced levels in the synapses that link cones to OFF-cone BCs. Thus, FAT3 is required to set up and/or maintain a properly organized synapse between photoreceptors and BCs and for the transmission of high-frequency signals, highlighting its multiple and versatile roles in retinal development and function.

## Materials and methods

### Animals

The *Fat3*^∆TM^ mouse line lacks exon 23, which contains the coding region for the transmembrane domain. Since no ICD anchored to the membrane has been detected (Avilés et al., 2022; Deans et al., 2011), this allele is expected to act as a full loss of function. The *Fat3*^floxed^ line contains LoxP sites flanking exon 23 (Deans et al., 2011). The *Fat3*^∆ICD-GFP^ mouse line has a deletion of most of the FAT3-ICD, which is replaced by GFP. This line possesses a full extracellular domain anchored to the cell membrane (Avilés et al., 2022). Heterozygous mice were used as breeders to obtain wildtype and knockout littermates. *Grik1*^−/−^ mice were obtained from Christophe Mulle (University of Bordeaux, Nouvelle-Aquitaine, France) (Mulle et al., 2000). *Grm6*^−/−^ mice (also known as *Grm6*^*nob3*^), which were characterized by Maddox and colleagues (Maddox et al., 2008), were purchased from The Jackson Laboratory (Strain #: 016883). *Ptprs^−/−^* mice were made by Michel Tremblay’s laboratory (McGill University, Montreal, Canada) (Elchebly et al., 1999). Transgenic mice expressing Cre recombinase were obtained from the following sources: *Ptf1a*^CRE^ (C. Wright, Vanderbilt University, Nashville, TN) (Fujitani et al., 2006); *Islet1*^CRE^ (a.k.a. *Isl1*^*tm1(cre)Sev*^/J) (Yang et al., 2006) (Strain #: 024242; The Jackson Laboratory); and *Bhlhe22*^CRE^ (a.k.a *Bhlhb5*^CRE^) (Ross et al., 2010) (M.E. Greenberg, Harvard Medical School). Mice were maintained on a 12/12-h light/dark cycle at 18–23°C and 40–60% humidity. Animals were handled ethically according to protocols approved by the Institutional Animal Care and Use Committee at Harvard Medical School. Randomization was used when selecting the animals to experiment with. Genotyping was done using real time PCR (Transnetyx).

### Electroretinography (ERG)

Mice were dark-adapted overnight before *in vivo* ERG recordings. Animals were anesthetized with 100/10 mg/kg ketamine/xylazine cocktail and placed on a heating pad. Their pupils were dilated with a drop of 1% tropicamide solution (Bausch + Lomb). Electrodes were applied to the cornea to pick up the electrical signals from the retina. Eyes were kept moist by a drop of phosphate-buffered saline (PBS). With an Espion E3 System (Diagonsys LLC), four types of ERG tests were performed: (1) scotopic test; (2) photopic tests with 1, 10, 100 and 1,000 cd s/m^2^ under a 30 cd/m^2^ background light to saturate the rod responses; (3) flicker tests at 0.5, 10, 20, 30, 40 and 50 Hz; and (4) step-light test with a 3-s light step of 1,000 cd/m^2^. The scotopic and photopic ERGs were conducted as described previously (Xiong et al., 2019). The flicker ERG was recorded using 3.162 cd s/m^2^ flashes as adapted from a published protocol (Tanimoto et al., 2015). The step ERG was created for this study to probe the d-wave from OFF-BCs. The experimenters were blinded to the genotypes of animals for ERG.

### Optical coherence tomography (OCT)

OCT of mouse eyes was conducted using an OCT2 system (Phoenix Research Labs) (Xiong et al., 2019). Briefly, anesthetized mice were pupil-dilated and placed on a heating pad to visualize the cross-section retina structure *in vivo* using spectrum domain OCT (light source: 160 nm ultrabroadband super-luminescent diode centered at 830 nm). 20 images were collected and averaged to generate a final OCT image. OCT imaging was performed on the mice *in vivo* immediately after the ERG tests to confirm the previously observed *ex vivo* histological changes. Before OCT imaging, a drop of GONAK 2.5% hypromellose solution (Akorn) was applied to the eye as the immersion medium with the OCT lens.

### Flicker-light–cued fear conditioning assay

**Figure S1.**
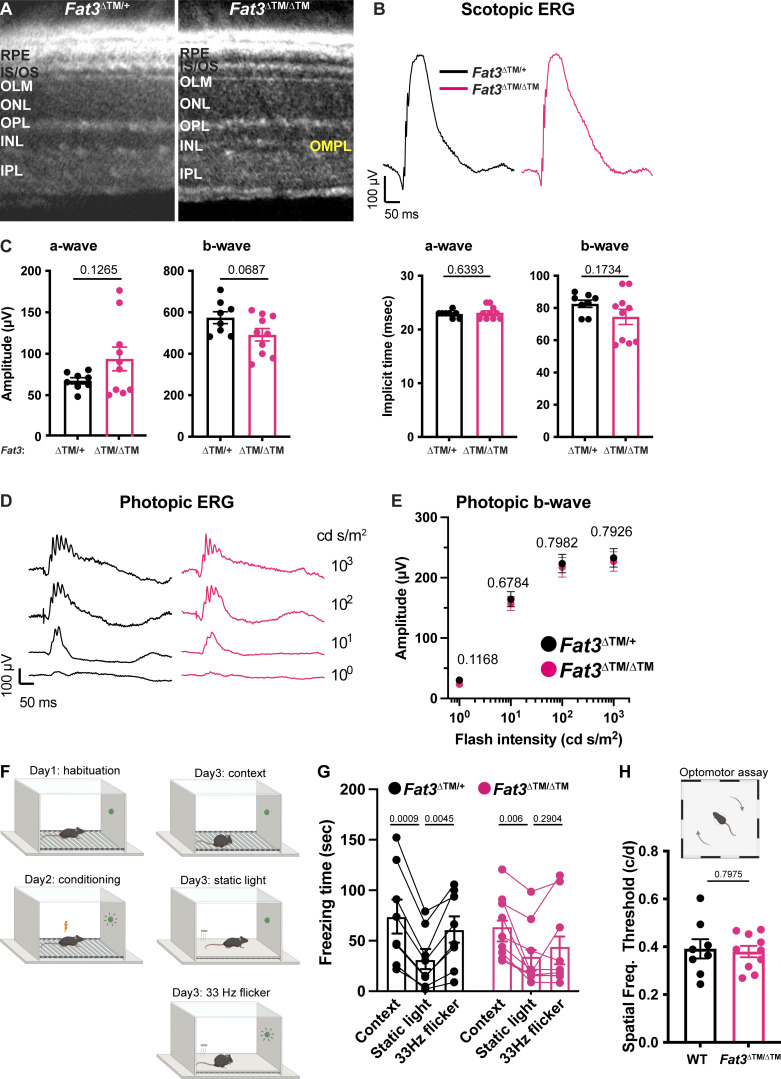
**Scotopic and photopic ERG of FAT3-deficient mice (Related to** Fig. 1 **). (A)** Representative OCT images of *Fat3*^∆TM/+^ control and *Fat3*^∆TM/∆TM^ eyes. **(B)** Representative scotopic ERG raw traces of *Fat3*^∆TM/+^ and *Fat3*^∆TM/∆TM^ eyes elicited by 0.1 cd s/m^2^ flashes. **(C)** Quantification of scotopic ERG parameters (amplitude and implicit time of a-wave and b-wave) of *Fat3*^∆TM/+^ (*n* = 8) and *Fat3*^∆TM/∆TM^ (*n* = 10) eyes. **(D)** Representative photopic ERG raw traces of *Fat3*^ΔTM^^/^+^^ and *Fat3*^ΔTM/ΔTM^ eyes elicited by 1, 10, 100, and 1,000 cd s/m^2^ flashes at 30 cd/m^2^ background light to saturate the response from rod-pathway. **(E)** Ensemble-averaged photopic ERG b-wave amplitude from *Fat3*^∆TM/+^ (*n* = 8) and *Fat3*^∆TM/∆TM^ (*n* = 10) eyes. **(F)** Schematics of fear conditioning and optomotor behavioral experiment. On Day 1, a mouse is brought to the electric-shock cage with a floor of metal bars for habituation of the environment. On Day 2, the mouse is conditioned by electrical shock paired with 33 Hz flashing light. On Day 3, the mouse is first subjected to a contextual check, in which the “Context” measures the freezing time of the mouse after it is brought back to the electric shock cage, which presents a fear-associated context environment, without the shock. “Static” measures the freezing time of the mouse with a static light, after the covering the metal bars and an odor change. Following this measurement, a 33 Hz flickering light is turned on, and the freezing time of the mouse is measured, as the “flicker” time. **(G)** Fear conditioning responses as freezing time (seconds) from *Fat3*^∆TM/+^ (*n* = 8) and *Fat3*^∆TM/∆TM^ (*n* = 9) mice. One-way ANOVA (matched data) with Dunnett multiple comparison test. **(H)** The visual threshold of spatial frequency of WT (*n* = 8) and *Fat3*^∆TM/∆TM^ (*n* = 10) mice measured with the optomotor behavioral assay shown in the bottom cartoon in panel D. Unpaired two-tailed Student’s *t* test. Abbreviations: RPE, retinal pigmented epithelium; IS/OS, inner-outer segments junction; OLM, outer limiting membrane; OPL, outer plexiform layer; IPL, inner plexiform layer; IMPL: inner misplaced plexiform layer. NS: non-significant. Error bars: SEM.

The fear conditioning test for high temporal frequency vision was created by modifying previously published protocols (Gaub et al., 2015). The Med Associates system was used for the tests with four LED lights (two green and two yellow) controlled by a computer software (Med PC). Mice were videotaped through Media Recorder Software, and the fear response of freezing was analyzed by researchers who were blinded to the genotypes as a surrogate measurement of memory linked with visual input. On Day 1, the mice were brought to the electric shock cage individually to get familiar with the environment and procedure. They were in the cage for 30 min under a dim house light in the background and LEDs were turned off (Fig. S1). On Day 2, mice were brought back to the electric shock cage, first exposed to static green/yellow LED lights for 2 min, followed by 30 s of 33 Hz LED flicker light (i.e., the cue). Within the last 2 s of the cue, a series of 0.7 mA shocks were initiated to trigger the fear memory linked with the cue. This static-flicker-shock cycle was repeated twice more. On Day 3, contextual memory (i.e., context test) was measured by placing the mouse back into the conditioning chamber for 3 min (no electric shock was delivered during this session), and the duration of freezing was recorded. Then, cued memory was measured by placing the mouse into an altered context, which was composed of different tactile and olfactory cues. The amount of freezing in the altered context was measured as a baseline (3 min, static light) followed by the measurement of freezing during presentation of the cued stimulus (3 min, 33 Hz flicker light). The videos were analyzed by an examiner blinded to the genotypes to extract the freezing time of all three conditions (i.e., context, static, and 33 Hz flicker).

### Optomotor assay

Using an OptoMotry System (CerebralMechanics), the optomotor assay to measure the visual acuity of mice was conducted as described previously (Xiong et al., 2019). The testing grates were set with 100% contrast and were moved at 1.5 Hz temporal frequency. The visual acuity (i.e., maximal spatial frequency in the unit of cycle/degree) was tested by an examiner, who was blinded to the genotype. During each testing episode, the examiner reported either “yes” or “no” to a computer program until the threshold of acuity was reached. The parameter of each testing episode (i.e., spatial frequency) was determined by the computer program and blinded to the examiner. The experimenters were blinded to the genotypes of animals being studied.

### Dissections and immunohistochemistry

Animals of the desired postnatal age (3 or 6 wk of age, as indicated) were euthanized by CO_2_ inhalation and cervical dislocation. Extraocular tissue, the cornea, and the lens were removed from the eyes and the eyecups were further fixed by immersion in 4% paraformaldehyde (PFA; Cat#15710; EMS) for 30 min (min) at room temperature or 15 min on ice. After several washes with PBS buffer, the eyes were submerged in 30% sucrose and kept at 4°C for at least 2 h. After sucrose cryoprotection, eyes were incubated in NEG-50 (Cat#84000-154; VWR) overnight at 4°C and embedded by freezing in a liquid nitrogen vapor bath. Retinal slices were obtained by cryosectioning the eyes at 20-µm thickness and mounting on Superfrost Plus Micro Slide (Cat#48311-703; VWR). The sections were either stained immediately or stored at −80°C.

For regular immunohistochemistry, NEG-50 was removed by short incubation in PBS and then sections were blocked and permeabilized by incubation in 5% normal donkey serum (NDS, Cat#017-000-121; Jackson ImmunoResearch) in Sorenson’s supplemented with 0.5% Triton-X for 1–2 h at room temperature. Sections were then incubated in primary antibody diluted in blocking buffer overnight at 4°C. After several washes with PBS, sections were incubated with fluorescent secondary antibodies diluted in 5% NDS in Sorenson’s buffer supplemented with 0.02% Triton-X for 1.5–2 h at room temperature. After final washes, sections were mounted in DAPI-Fluoromount-G (Cat#0100-20; SouthernBiotech). Primary antibodies (also see Table 1) used for immunohistochemistry were as follows: rabbit anti-ARR3 (Cat#AB15282; Millipore Sigma), goat anti-Bhlhb5 (1:500; Cat#sc-6045; Santa Cruz Biotechnology), mouse anti-CtBP2 (1:2,000, Cat#612044; BD Biosciences), rabbit anti-dsRed (cross-reacts with TdTomato, 1:1,000, Cat#632496; Clontech), mouse anti-FAT3 (Avilés et al., 2022; Deans et al., 2011) (1:200), chicken anti-GFP (1:500; Cat#GFP-1020; Aves), mouse anti-GRIK1 (GluR5, 1:200, Cat#sc-393420; Santa Cruz Biotechnology) (Nemitz et al., 2021), sheep anti-GRM6 (1:2,000, a gift from Jeannie Chen, University of Southern California, Los Angeles, CA, USA, and originally developed by Kirill Martemyanov Lab [Cao et al., 2009]), goat anti-PTPσ (1:200; Cat# AF3430; R&D Systems), rabbit anti-VGAT (1:300; Cat#131002; SynapticSystems), and mouse anti-VSX2 (Chx10, 1:100; Cat#sc-365519; Santa Cruz Biotechnology). All secondary antibodies were diluted 1:1,000 and were Donkey anti-chicken Alexa Fluor 488, donkey anti-goat Alexa Fluor 568, donkey anti-mouse Alexa Fluor 488, donkey anti-mouse Alexa Fluor 568, goat anti-mouse Alexa Fluor 647, donkey anti-rabbit Alexa Fluor 488, donkey anti-rabbit Alexa Fluor 568, donkey anti-rabbit Alexa Fluor 647, and donkey anti-sheep Alexa Fluor 568.

**Table 1.** Key resources table

Reagent or resource	Source	Identifier
**Antibodies**		
Rabbit anti-ARR3	Millipore Sigma	Cat#AB15282; RRID:AB_ 1163387
Goat anti-Bhlhb5	Santa Cruz	Cat#sc-6045; RRID:AB_2065343
Mouse anti-CTBP2	BD Biosciences	Cat#612044; RRID:AB_399431
Rabbit anti-dsRed	Clontech	Cat#632496; RRID:AB_10013483
Mouse anti-FAT3	Avilés et al. (2022), Deans et al. (2011)	N/A; RRID:AB_2904260
Chicken anti-GFP	Aves	Cat#GFP-1020; RRID:AB_10000240
Mouse anti-GRIK1 (GluR5)	Santa Cruz Biotechnology	Cat#sc-393420; RRID:AB_2716684
Sheep anti-GRM6	Cao et al. (2009)	N/A
Goat anti-PTPσ	R&D Systems	Cat#AF3430; RRID:AB_2175157
Mouse anti-PTPσ	Medimabs	Cat#MM-0020-P
Rabbit anti-VGAT	Synaptic systems	Cat#131002; RRID:AB_887871
Mouse anti-VSX2 (Chx10)	Santa Cruz Biotechnology	Cat#sc-365519; RRID:AB_10842442
Donkey anti-chicken, Alexa Fluor 488	Jackson ImmunoResearch	Cat#703-545-155; RRID:AB_2340375
Donkey anti-goat, Alexa Fluor 568	Thermo Fisher Scientific	Cat#A11057; RRID:AB_142581
Donkey anti-mouse, Alexa Fluor 488	Abcam	Cat#ab150105; RRID:AB_2732856
Donkey anti-mouse, Alexa Fluor 568	Thermo Fisher Scientific	Cat#A10037; RRID:AB_2534013
Goat anti-mouse, Alexa Fluor 647	Thermo Fisher Scientific	Cat#A-21235; RRID:AB_2535804
Donkey anti-rabbit, Alexa Fluor 488	Thermo Fisher Scientific	Cat#A21206; RRID:AB_2535792
Donkey anti-rabbit, Alexa Fluor 568	Thermo Fisher Scientific	Cat#A10042; RRID:AB_2534017
Donkey anti-rabbit, Alexa Fluor 647	Thermo Fisher Scientific	Cat#A31573; RRID:AB_2536183
Donkey anti-sheep, Alexa Fluor 568	Thermo Fisher Scientific	Cat#A-21099; RRID:AB_2535753
Goat anti-mouse – HRP	BioRad	Cat#170-6516; RRID:AB_11125547
**Commercial assays**		
RNAscope Multiplex Fluorescent Reagent Kit v2	ACD	Cat#323100
HCR IHC Bundle	Molecular Instruments	N/A
**Experimental models: Organisms/strains**		
Mouse: *Ptf1a*^CRE^	C. Wright, Vanderbilt U (Fujitani et al., 2006)	MGI:2387812
Mouse: *Isl1*^CRE^	The Jackson Laboratory (Yang et al., 2006)	Strain #: 024242
Mouse: *Bhlhe22*^CRE^	M.E. Greenberg (Harvard Medical School) (Ross et al., 2010)	N/A
Mouse: *Fat3*^floxed^	Deans et al. (2011)	N/A
Mouse: *Fat3*^∆TM^	Deans et al. (2011)	N/A
Mouse: *Fat3*^∆ICD-GFP^	Avilés et al. (2022)	N/A
Mouse: *Grik1*^−/−^	Christophe Mulle (University of Bordeaux) (Mulle et al., 2000)	N/A
Mouse: *Grm6*^−/−^	The Jackson Laboratory (Maddox et al., 2008)	Strain #: 016883
Mouse: *Ptprs*^−/−^	Michel Tremblay (Elchebly et al., 1999)	MGI:2158757
***In situ* hybridization probes**		
RNAscope Probe- Mm-*Fat3*-O1	ACD	Cat#509051
RNAscope Probe- Mm-*Grik1*-C3	ACD	Cat#438771-C3
**Recombinant DNA**		
AAV-Grik1-GFP plasmid	This paper	N/A
**Viral vectors**		
AAV8-Grik1-GFP	This paper	N/A

To detect FAT3 on retinal sections, we performed Hybridization Chain Reaction Immunohistochemistry (HCR-IHC) (Schwarzkopf et al., 2021) according to the manufacturer’s instructions (Molecular Instruments). In brief, on Day 1, sections were treated similarly to regular immunohistochemistry. After overnight incubation of the primary mouse anti-FAT3 (Deans et al., 2011) (1:200) antibody, sections were rinsed with PBS-0.1% Tween-20 (PBS-T) and incubated with 1 µg/ml of initiator-labeled anti-mouse secondary antibody (Molecular Instruments) for 1 h at room temperature. Slides were rinsed with PBS-T and a final rinse with 5X Saline-Sodium Citrate buffer with 0.1% Tween-20 (SSC-T) and incubated with amplification buffer (Molecular Instruments) for 30 min at room temperature. H1 and h2 fluorescently labeled hairpins were separately denaturated at 95°C for 90 s followed by 30 min incubation at room temperature in the dark. A 60-mM hairpin solution mix was prepared by adding snap-cooled h1 and h2 hairpins to the amplification buffer and incubated on the slides over night at room temperature in a dark, humidified chamber. After several washes with SSC-T, sections were mounted with DAPI-Fluoromount-G.

### *In situ* hybridization (RNAscope)

Tissue collection was performed similar to immunohistochemistry, except using RNAase-free conditions. For RNAscope *in situ* hybridization, we used RNAscope Fluorescent Multiplex Reagent Kit v2 (Cat#323120; ACD) assay following the manufacturer’s instructions. In brief, retinal sections were post-fixed in 4% PFA for 15 min at room temperature, treated with hydrogen peroxide for 10 min at room temperature and treated with Protease III for 10 min at 40°C before probe incubation. Probes were obtained from ACD (see Table 1). Immunohistochemistry was performed after *in situ* hybridization by rinsing the sections in PBS after the final RNAscope wash and permeabilized and blocked again with 5% NDS/0.5% Triton X-100 Sorenson’s buffer, followed by the regular immunohistochemistry protocol.

### *In vivo* viral injection

The AAV-Grik1-GFP plasmid was generated by cloning a previously identified Grik1 enhancer (CRM4) (Kishi et al., 2019) upstream of a simian virus 40 (SV40) intron, Kozak sequence, GFP coding sequence, woodchuck hepatitis virus posttranscriptional regulatory element (WPRE), and polyadenylation sequence. To produce the AAV8-Grik1-GFP vector, HEK293T cells were triple-transfected with a mixture of AAV-Grik1-GFP plasmid, adenovirus helper plasmid, and rep2/cap8 packaging plasmid. Viral particles were harvested from the supernatant 72 h after transfection and purified using an iodixanol gradient as described previously (Grieger et al., 2006). The titer of AAV8-Grik1-GFP was determined by comparing SYPRO Ruby (Molecular Probes) staining for viral capsid proteins (VP1, VP2, and VP3) to that of a reference vector with a known titer.

To deliver AAV8-Grik1-GFP into the developing retina, we injected it into the subretinal space as described previously (Matsuda and Cepko, 2004; Wang et al., 2014). In brief, neonatal P2–3 mouse pups were anesthetized by chilling on ice. We injected 2.5 × 10^9^ vector genomes (vg) per eye, which is at a titer not toxic to the eye, diluted in PBS and 0.1% Fast Green (for visualization) using a pulled borosilicate glass needle with an opening of 0.5–1 mm diameter connected to an Eppendorf FemtoJet injector into the subretinal space. The pups recovered on a warm pad, and upon regaining consciousness they were returned to their mother. We then let them develop until performing histological procedures at P22.

### Image acquisition

After immunohistochemistry or RNAscope, retinal sections were imaged within 300 µm from the optic nerve head on a Leica SP8 or a Zeiss LSM800 confocal microscope, using the LAS X or the ZEN lite software, respectively. The entire sections were imaged at room temperature in consecutive z-slices separated by 1 µm using a 40×/1.3 or 63×/1.4 objective with immersion oil. Fluorochromes Alexa Fluor 488, Alexa Fluor 568, Alexa Fluor 647, and DAPI were excited with lasers and their emitted fluorescence was detected using the appropriate wavelengths. The z stacks were then projected at maximum fluorescence intensity using Fiji/ImageJ (Schneider et al., 2012; Schindelin et al., 2012).

### Histochemical quantifications

We assigned random numbers to each image to ensure blinded quantifications. Only after the quantification was done, the identity of the images was revealed to assign the values to their corresponding genotype. All the procedures for independent experiments were done under the same technical parameters, and the comparisons were made between control and experimental conditions within the same experiment to avoid batch effects. The animal number (*n*) and biological replicates (i.e., sections) number, statistical test performed, and P values are indicated in figure legends and/or figures.

We assessed AC migration and the “ectopic synapse score” or “OMPL score” as described previously (Avilés et al., 2022). To quantify expression of PTPσ, GRIK1, or GRM6 proteins in the OPL, the images were thresholded until background signal in the OPL was not observed. All images from the same experiment were treated the same way using Fiji (ImageJ). Then, the integrated density was measured to quantify protein expression in the same total area of each image on the OPL region (44.69 × 17.78 µm). To quantify CtBP2 fluorescence intensity on immunohistochemistry samples, we used Fiji (ImageJ) to measure the mean gray value on areas of the OPL by tracing a rectangle that took up most of the OPL height. In addition, the mean gray value was measured on a rectangle traced on the ONL (region where photoreceptors reside and is used as background signal) to normalize the value of the OPL.

For *in situ* hybridization analysis, both the cells (as defined by VSX2 staining) and mRNA were segmented using average diameters by using CellProfiler to define their boundaries. Fluorescence intensity was measured in the *Grik1* and *Grm6* channels, and the corresponding values were quantified for each cell to calculate the overall averages.

### Statistics

To determine significant differences between control and experimental groups, we used Prism10 software (GraphPad Software Inc.) for statistical analysis. We used a nested analysis to calculate the P values for histological data. For ERG data, we applied Student’s *t* test, or if more than two groups, one-way ANOVA (matched) with Dunnett multiple comparison test.

### GST pull down and western blot

For binding analysis, we performed western blots of supernatants after pulling down binding partners from mouse brain protein homogenates with the FAT3-ICD fused to Glutathione-S-transferase (GST) and GST alone generated previously (Avilés et al., 2022). Samples were denatured at 95°C for 10 min and subjected to SDS-PAGE in a 4–12% Criterion XT Bis-Tris Protein Gel (Bio-Rad) using XT MES Running Buffer (Bio-Rad). After 2 h at 150 V of electrophoresis, the proteins were transferred to Immobilon-P PVSF (0.45 µm; Sigma-Millipore) in Tris-Glycine buffer supplemented with 20% methanol for 1 h at 75 V. The Immobilon-P membranes were blocked with 5% skim milk in TBS buffer and then incubated with primary antibodies at 4°C overnight. The primary antibody used for western blots was mouse anti-PTPσ (1:1,000, Cat# MM-0020-P; Medimabs). After several washes with TBS supplemented with 0.5% Tween 20 (Sigma-Aldrich), the membranes were incubated with a secondary goat anti-mouse HRP antibody (Cat# 170-6516; Biorad) diluted 1:2,000 for 1–2 h at room temperature. The signal was developed using Clarity ECL substrate following the manufacturer’s instructions (Bio-Rad). Western blots were done at least twice with similar results.

### External gene expression profile datasets

Gene expression in different types of retinal BCs was analyzed by using the single-cell RNAseq database (https://singlecell.broadinstitute.org/single_cell/study/SCP3/retinal-bipolar-neuron-drop-seq#study-visualize) and a modified R script that was published previously (Shekhar et al., 2016).

### Online supplemental material

Figs. S1, S2, S3, S4, S5, and S6 are provided as supplemental figures related to Figs. 1, 2, 3, 4, 6. and 7, respectively. Source data underlying all figures is provided as Data S1 in MS Excel format.

## Results

### Global loss of *Fat3* affects the ERG response to high temporal frequency light

In *Fat3*^ΔTM/ΔTM^ mutant mice, which lack a membrane-localized form of FAT3, retinal lamination is strongly disrupted, with changes in the position of ACs and their synapses with other ACs, as well as with other retinal cell types (Deans et al., 2011). The abnormal lamination can also be seen by OCT imaging in animals *in vivo* (Fig. S1 A). To assess the functional consequences of this change in retinal cell organization, we used the ERG, a common way to measure electrical changes in the retina in response to light. By altering the stimulus, it is possible to reveal the contributions of specific retinal pathways. For instance, signaling through the rod pathway is assayed by performing the ERG after dark-adaptation and under scotopic conditions using dim flashes that elicit little response from the cone pathway. Conversely, photopic ERG tests isolate the cone pathway by light-adapting the eyes and using a background light that saturates rod phototransduction. In an ERG waveform, the a-wave originates from photoreceptors and is followed by the b-wave, which reflects the activity of rod bipolar cells (RBC) and/or ON-types of cone BCs (ON-CBCs). By flashing the stimulus on and off with increasing frequency, it is possible to determine how well photoreceptors and BCs are able to resolve temporal differences in the visual stimulus. As the frequency of the flash increases, OFF-CBCs are hypothesized to dominate the response (Tanimoto et al., 2015). Additionally, the ERG d-wave, which emerges when turning off a light step that lasts for a few seconds (Stockton and Slaughter, 1989), is thought to represent mainly OFF-CBC activity (Xu and Karwoski, 1995). To characterize the temporal tuning of specific OFF-CBC subtypes, patch clamping of individual cells has been done with flickers up to 21 Hz *ex vivo* (Ichinose and Hellmer, 2016). Our study aims to characterize the overall resolution and threshold of temporal vision *in vivo* using ERG, as we are interested in how FAT3 might contribute to vision and behavior, particularly in light of the abnormal lamination patterns observed in FAT3 mutants.

Conventional ERG assays under scotopic and photopic conditions revealed no obvious difference in *Fat3*^ΔTM/ΔTM^ versus *Fat3*^ΔTM/+^ littermates, indicating that overall signaling through rod and cone pathways was preserved (Fig. S1, B–E). Likewise, the oscillatory potentials between the a- and b-waves, which are thought to reflect the activity of ACs and/or RGCs (Kolb et al., 1995; Liao et al., 2023), showed no dramatic change in the number, amplitude, or timing of peaks (Fig. 1, B–D). Thus, *Fat3*^ΔTM/ΔTM^ mutant mice can detect standard light stimuli despite abnormal retinal lamination.

Although previous work focused on its role in ACs (Avilés et al., 2022; Deans et al., 2011), *Fat3* is also expressed in RGCs and some subtypes of BCs, including several subtypes of OFF-CBCs (Shekhar et al., 2016) implicated in responses to lights that flicker in the high-frequency range (i.e., over 15 Hz [Tanimoto et al., 2015]). To determine whether *Fat3* is required for the proper response to high temporal frequency light, we recorded ERGs in the range hypothesized to be dominated by the OFF-pathway (Tanimoto et al., 2015). We observed a significant decrease in the amplitude of responses to lights flickering at 30–50 Hz in *Fat3*^ΔTM/ΔTM^ compared with their *Fat3*^ΔTM/+^ littermates (Fig. 1, E and F). Additionally, the timing of the first peak in response to 20 Hz stimulation (“the implicit time”) was delayed (Fig. 1 G). The >30 Hz implicit time was not measured, as many *Fat3* mutant mice presented no response at this frequency. These results suggested that the transmission of high temporal frequency light responses was impaired, presumably in BCs, where the signal to the flicker ERG likely originates (Tanimoto et al., 2015).

To determine if this physiological deficit in the retina had perceptual consequences, behavioral assays were conducted to examine if the mutant mice could perceive high-frequency flickering light using contextual and vision-cued fear conditioning tests (Gaub et al., 2015; Shi et al., 2015). Normally, fear-conditioned animals increase the time of “freezing” if they have been conditioned to associate an unpleasant stimulus with an environmental cue, in this case, 33 Hz light (Fig. S1, F and G). In contrast to *Fat3*^ΔTM/+^ mice (eight out of eight animals), significantly fewer *Fat3*^ΔTM/ΔTM^ mice (four out of nine animals) showed increased freezing response when switching from a static light to a 33 Hz flicker (Fig. S1 G). This was similar to the 30 Hz flicker ERG results where only 4 out of 10 *Fat3*^ΔTM/ΔTM^ eyes presented amplitudes >20 μV, which is the level at which noise can be distinguished from signal and is thus the empirically determined resolution of ERG recordings (Fig. 1 F). The lack of response was not due to an inability to form fear memories, as all *Fat3*^ΔTM/ΔTM^ and *Fat3*^ΔTM/+^ animals froze less when switching from an unpleasant olfactory and tactile context (group “context”) to a novel and safe context within the group with static light (group “static light”) (Fig. S1 G). Mutant mice also performed like the wildtype (WT) controls in an optomotor behavioral assay, which measures spatial discrimination (Fig. S1 H).

Responses to different frequencies of light are used to study the temporal properties of vision at photoreceptor, brain, or psychophysical levels (Donner, 2021). The flicker ERG at high temporal frequency has been proposed to depend on the OFF-CBC function (Tanimoto et al., 2015). OFF-CBC function can be probed at the single cell level using patch-clamping or at the population level by examination of the d-wave of the ERG (Stockton and Slaughter, 1989), which has been used with amphibian retina *ex vivo* and the primate retina *in vivo* (Sieving et al., 1994; Xu and Karwoski, 1995). To our knowledge, there are no reports of an ERG protocol to measure the d-wave from mice *in vivo*. To address this unmet need, we designed an *in vivo* ERG protocol for mice based on a study of the *ex vivo* ERG response of amphibian retinas (Stockton and Slaughter, 1989). This assay measures the retinal voltage change, the d-wave, after turning off a long step of light (i.e., light-off) (Stockton and Slaughter, 1989), which is called a “step ERG.” Using this *in vivo* protocol on mice, we found that WT mice had a strong d-wave with robust oscillatory potentials at the end of a three-second exposure to a 1,000 cd/m^2^ step of light (Fig. 1, H and I). By contrast, in *Fat3*^ΔTM/ΔTM^ mice, the d-wave amplitude was decreased and the d-wave related oscillatory potentials were absent (Fig. 1, H and I). Thus, responses to both high-frequency flickering light and light-off stimuli showed that *Fat3*^ΔTM/ΔTM^ mice have deficits in processing specific types of visual stimuli that have been associated with OFF-CBCs.

**Figure 2.**
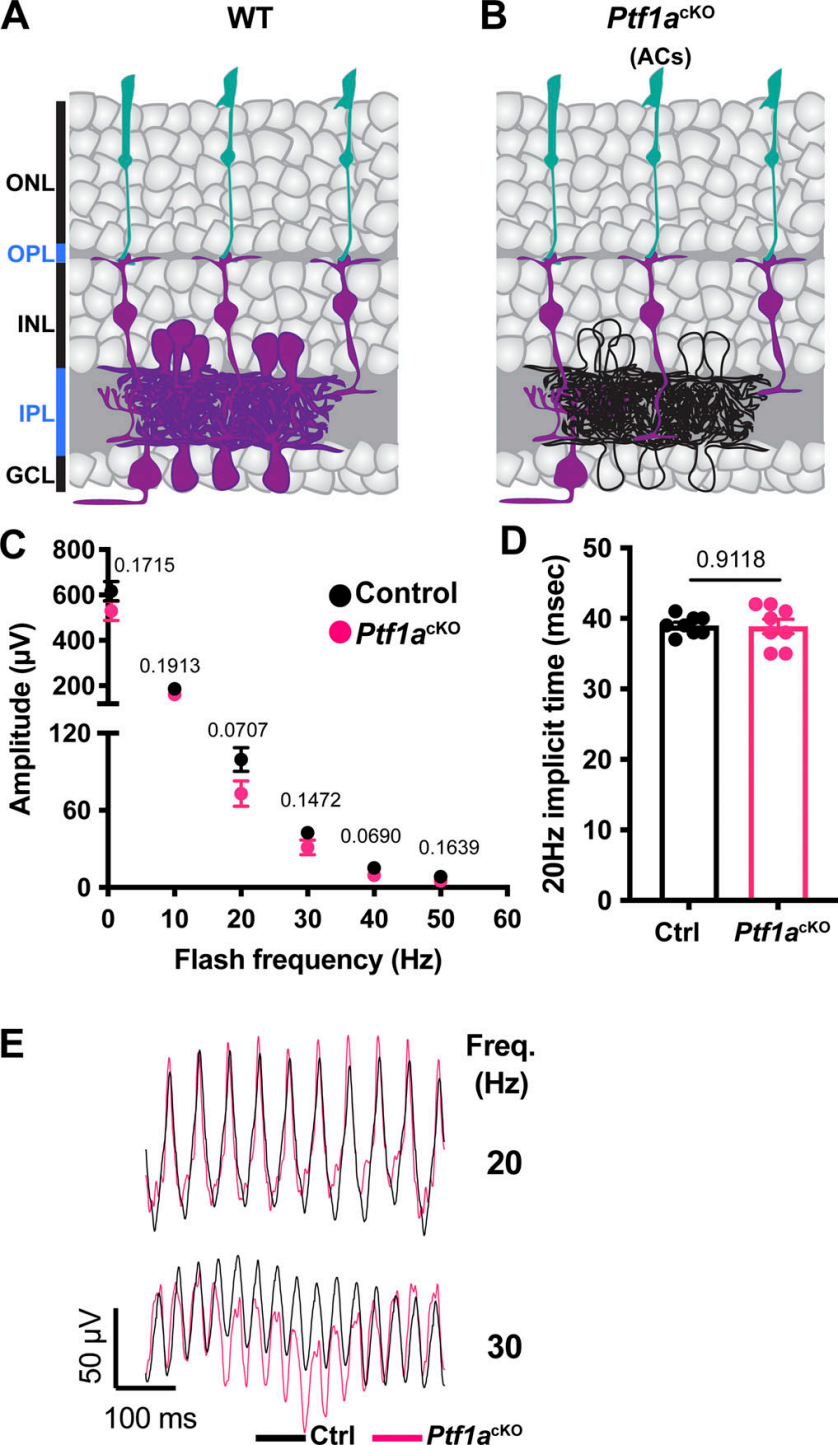
**Flicker ERG at high frequency of *Ptf1a*** ^**CRE**^ **conditional *Fat3* mice (*Ptf1a*** ^**cKO**^ **). (A)** Schematic representation of cell classes that express FAT3 in wild type retina, shown in magenta. **(B)** Schematic representation of cell classes, i.e., ACs, that lose *Fat3* expression in a *Ptf1a*^*cKO*^, shown in black outlines. **(C)** Flicker ERG amplitude at 0.5, 10, 20, 30, 40, and 50 Hz for the *Ptf1a*^*cKO*^ condition. Control exact genotypes are *Ptf1a*^CRE^;*Fat3*^fl/+^ (*n* = 8 eyes) and *Ptf1a*^*cKO*^ exact genotypes are *Ptf1a*^CRE^;*Fat3*^fl/∆TM^ (*n* = 8 eyes). Unpaired two-tailed Student’s *t* test. Error bar: SEM. **(D)** Flicker ERG implicit time at 20 Hz for control (39.0 ± 0.4 ms, *n* = 8 eyes) and *Ptf1a*^cKO^ (38.9 ± 1.0 ms, *n* = 8 eyes). Unpaired two-tailed Student’s *t* test. Error bar: SEM. **(E)** Representative flicker ERG raw traces for control (*n* = 8 eyes) and *Ptf1a*^cKO^ (*n* = 8 eyes).

To test the impact of an ectopic plexiform layer and other FAT3-dependent AC properties on retinal physiology, *Fat3* was deleted specifically from ACs using *Ptf1a*^CRE^ (Deans et al., 2011; Fujitani et al., 2006) and a *Fat3*-floxed allele (Fig. 2, A and B). In these conditional knock-out (*Ptf1a*^cKO^) mutants, ACs migrate normally but do not retract their trailing processes, leading to the formation of an OMPL (Deans et al., 2011). We found that >30 Hz flicker ERG amplitude and 20 Hz implicit time were normal in *Ptf1a*^cKO^ mice compared with littermate control mice (Fig. 2, C–E), indicating that the high-frequency light response defects were not secondary either to the presence of *Fat3* mutant ACs or the mild lamination defects they caused. This raised the possibility that the highly specific physiological deficits seen in *Fat3*^ΔTM/ΔTM^ mice are due instead to altered BC function.

**Figure 3.**
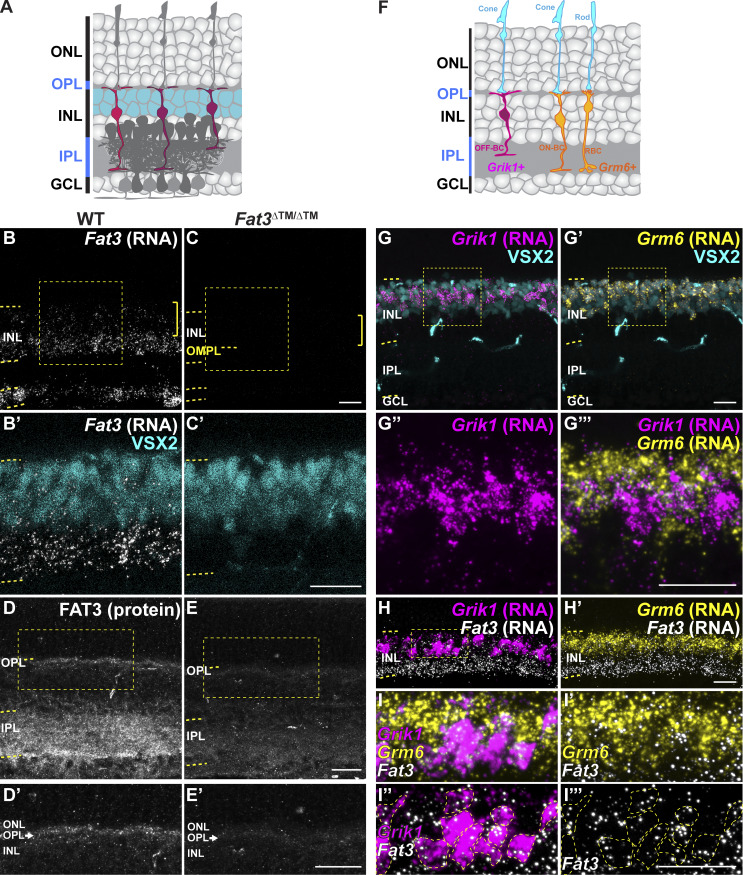
***Fat3* RNA is enriched in OFF-cone bipolar cells. (A)** Schematic representation of the retina highlighting the position of bipolar cell bodies, as stained by VSX2. **(B)***In situ* hybridization for *Fat3* RNA in WT P22 retinas. **(C)***In situ* hybridization for *Fat3* RNA in *Fat3*^∆TM/∆TM^ P22 retinal tissue. **(B′ and C′)** In B and C the RNA puncta are shown in white and the yellow brackets indicate the area of VSX2^+^ cell bodies (cyan, B′ and C′). Yellow dashed lines demark the inner nuclear layer (INL) and the outer misplaced plexiform layer (OMPL) in *Fat3*^∆TM/∆TM^ tissue. The squares demark the insets seen in B′ and C′ at higher magnification. VSX2 protein is seen in cyan. **(D)** Hybridization chain reaction-immunohistochemistry (HCR-IHC) of FAT3 in wild type retinas. **(D′)** Inset demarked in a yellow box in D is shown at higher magnification in D′. **(E)** HCR-IHC of FAT3 in *Fat3*^∆TM/∆TM^ mutant retinas. **(E′)** Inset demarked in a yellow box in E is shown at higher magnification in E′. **(F)** Schematic representation of *Grik1* and *Grm6* RNA enrichment in bipolar cells, according to data in Fig. S2. **(G and G′)***in situ* hybridization of *Grik1* RNA (magenta) and *Grm6* RNA (yellow, G′) with immunostaining for VSX2 (cyan). **(G″ and G‴)** The insets in G and G′ are shown at a higher magnification in G″ and G‴. **(H)** Triple *in situ* hybridization of *Fat3* (white), *Grik1* (magenta), and *Grm6* (yellow) RNA. **(I–I‴)** Inset in H is seen at higher magnification in I–I‴. **(I)** Higher magnification of inset shown in H. Yellow dashed lines in I″ and I‴ demark *Grik1* RNA^+^ cell bodies. *Fat3* RNA (white) is shown together with *Grm6* (yellow) RNA in I′, with *Grik1* (magenta) RNA in I″ and alone in I‴. Scale bars: 20 µm.

**Figure S2.**
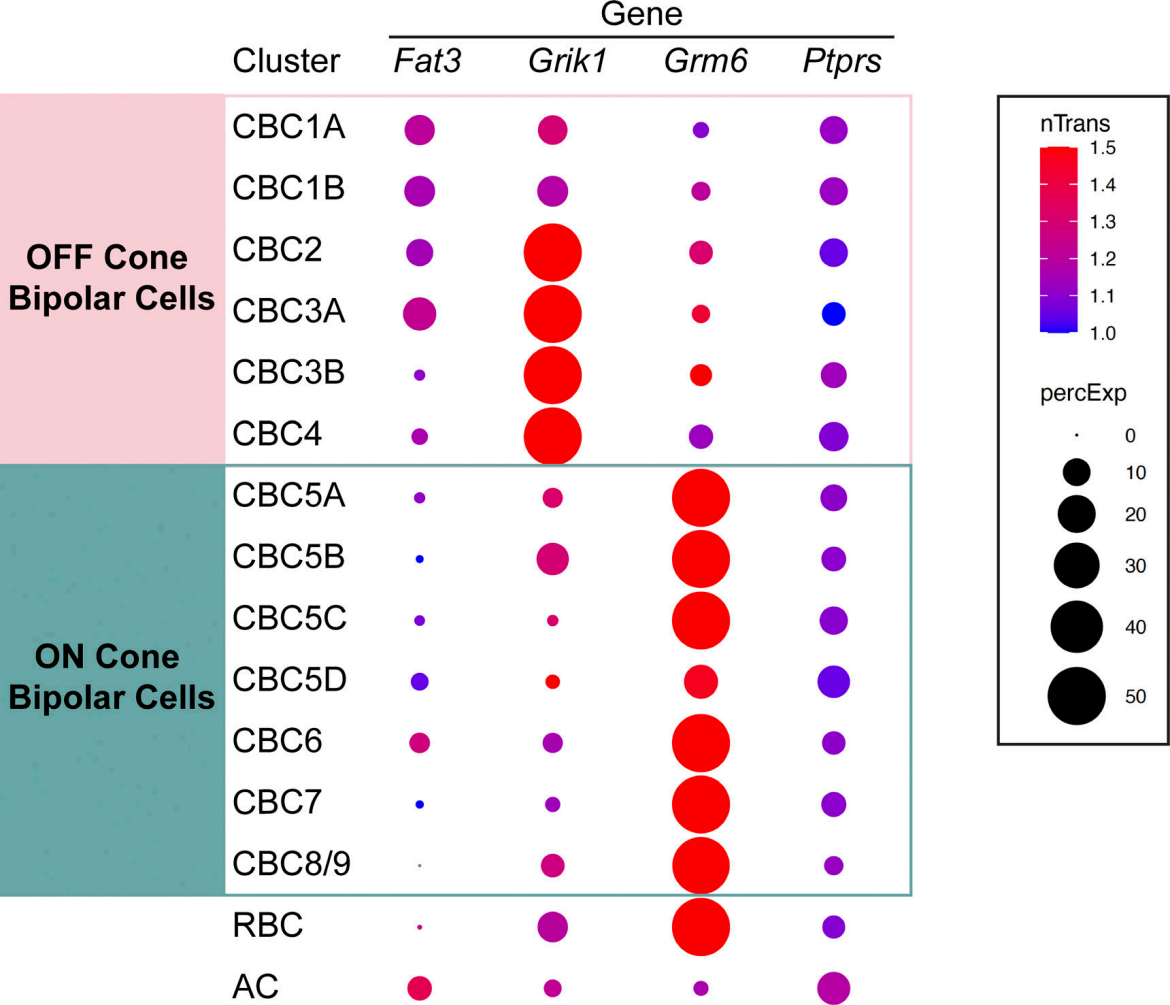
**scRNAseq data display (Related to** Fig. 2 **).** Expression of *Fat3*, *Grik1*, *Grm6*, and *Ptprs* gene transcripts by bipolar cell identity are displayed. These data are based on Shekhar et al. (2016).

Consistent with previous single-cell RNA sequencing data showing its expression in type 1A, 1B, 2, and 3A CBCs (Shekhar et al., 2016), we confirmed that *Fat3* mRNA is present in BCs by *in situ* hybridization (Fig. 3, A–C). Additionally, FAT3 protein was detected not only in the IPL but also in the OPL, where BC dendrites form synapses with photoreceptors (Fig. 3 D). In situ hybridization confirmed co-expression of *Fat3* with the OFF-CBC marker *Grik1*, which encodes an ionotropic glutamate receptor (Shekhar et al., 2016; Kishi et al., 2019; Borghuis et al., 2014; DeVries, 2000; Haverkamp et al., 2001, 2003; Ichinose and Hellmer, 2016; Puller et al., 2013; Puthussery et al., 2014) (Fig. 3, F–I). *Fat3* is also expressed in some *Grik1*-negative CBCs, which are positive for the ON-CBC marker *Grm6*, though to a lesser degree (Fig. 3 I and Fig. S2). This expression pattern is in line with FAT3 having a role on retinal functions associated with OFF-CBC pathways, as was suggested by the flicker and step ERG responses.

### Retinal lamination defects do not disrupt ERG responses to flickering stimuli

**Figure 4.**
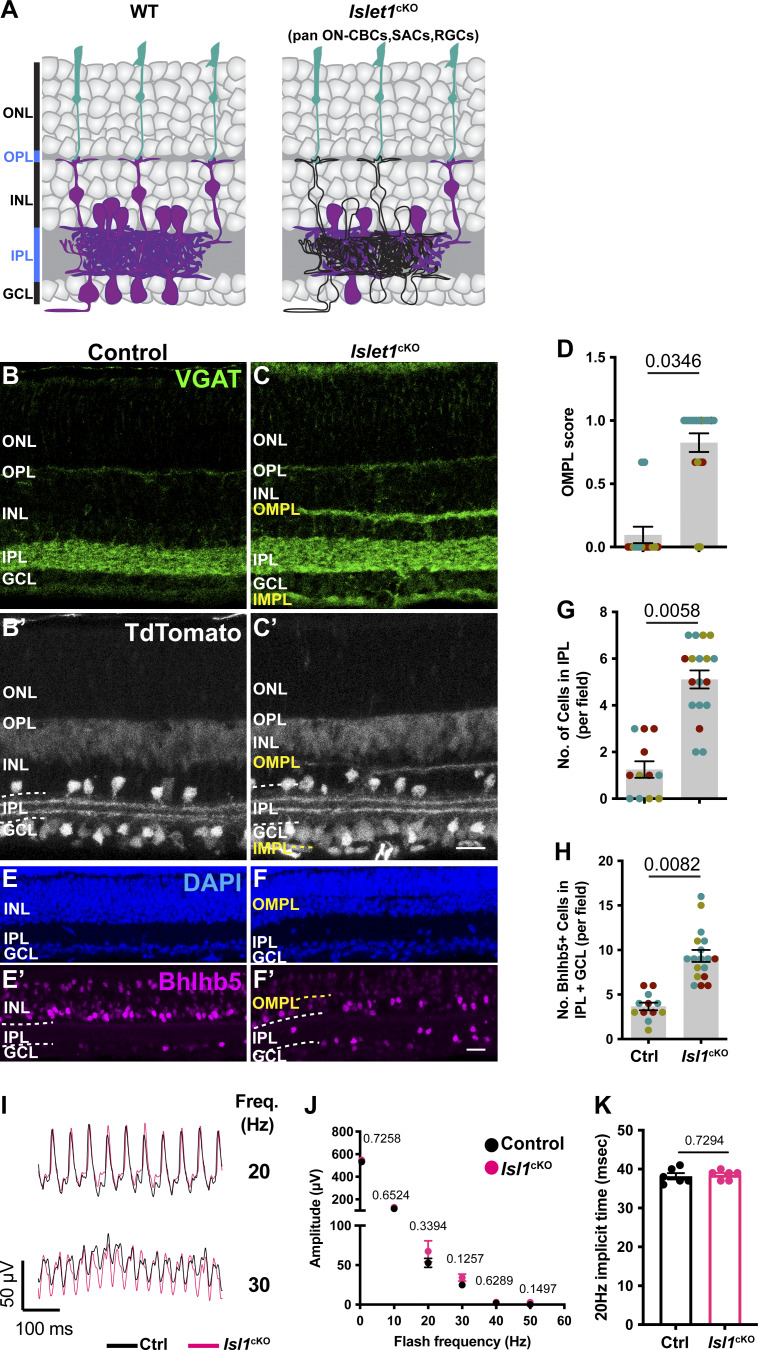
**Flicker ERG at high frequency of *Isl1*** ^**CRE**^ **conditional *Fat3* mice (*Isl1*** ^**cKO**^ **). (A)** Schematic representation of cell classes, i.e., starburst ACs, RGCs, and ON-CBCs, that lose *Fat3* expression in an *Isl1*^cKO^ shown in black outlines compared to magenta cells seen in WT controls. **(B)** VGAT immunostaining for control (exact genotype throughout this figure: *Isl1*^CRE/+^;*Fat3*^fl/+^) mice. **(C)** VGAT immunostaining for *Isl1*^cKO^ (exact genotype throughout this figure: *Isl1*^CRE/+^;*Fat3*^fl/∆TM^). **(B′ and C′)** TdTomato reporter of Cre expression is seen in B′ and C′. **(D)** Quantification of the OMPL score for *Isl1*^cKO^. Controls: 0.095 ± 0.065 (*n* = 3 animals, 14 retinal regions); *Isl1*^cKO^: 0.825 ± 0.074 (*n* = 3 animals, 19 retinal regions). Each data point corresponds to a retinal region, color-coded by animal, nested two-tailed test. Error bar: SEM. **(E)** DAPI and Bhlhb5 immunostaining of control retinas. **(F)** DAPI and Bhlhb5 immunostaining of *Isl1*^cKO^ retinas. **(G)** Quantification of the number of nuclei per field in the IPL. Controls: 1.25 ± 0.35 (*n* = 3 animals, 12 retinal regions); *Isl1*^cKO^: 5.11 ± 0.39 (*n* = 3 animals, 18 retinal regions). Each data point corresponds to a retinal region, color-coded by animal, nested two-tailed test. Error bar: SEM. **(H)** Quantification of the number of Bhlhb5^+^ nuclei per field in the IPL and GCL. Controls: 3.67 ± 0.43 (*n* = 3 animals, 12 retinal regions); *Isl1*^cKO^: 9.33 ± 0.67 (*n* = 3 animals, 18 retinal regions). Each data point corresponds to a retinal region, color-coded by animal, nested two-tailed test. Error bar: SEM. **(I)** Representative flicker ERG raw traces for control and *Isl1*^cKO^. **(J)** Flicker ERG amplitude at 0.5, 10, 20, 30, 40, and 50 Hz for control (*n* = 6 eyes) and *Isl1*^cKO^ (*n* = 6 eyes). Unpaired two-tailed Student’s *t* test. Error bar: SEM. **(K)** Flicker ERG implicit time at 20 Hz for control (38.1 ± 0.8 ms, *n* = 6 eyes) and *Isl1*^cKO^ (38.5 ± 0.5 ms, *n* = 6 eyes). Unpaired two-tailed Student’s *t* test. Error bar: SEM. Scale bars: 20 µm.

**Figure S3.**
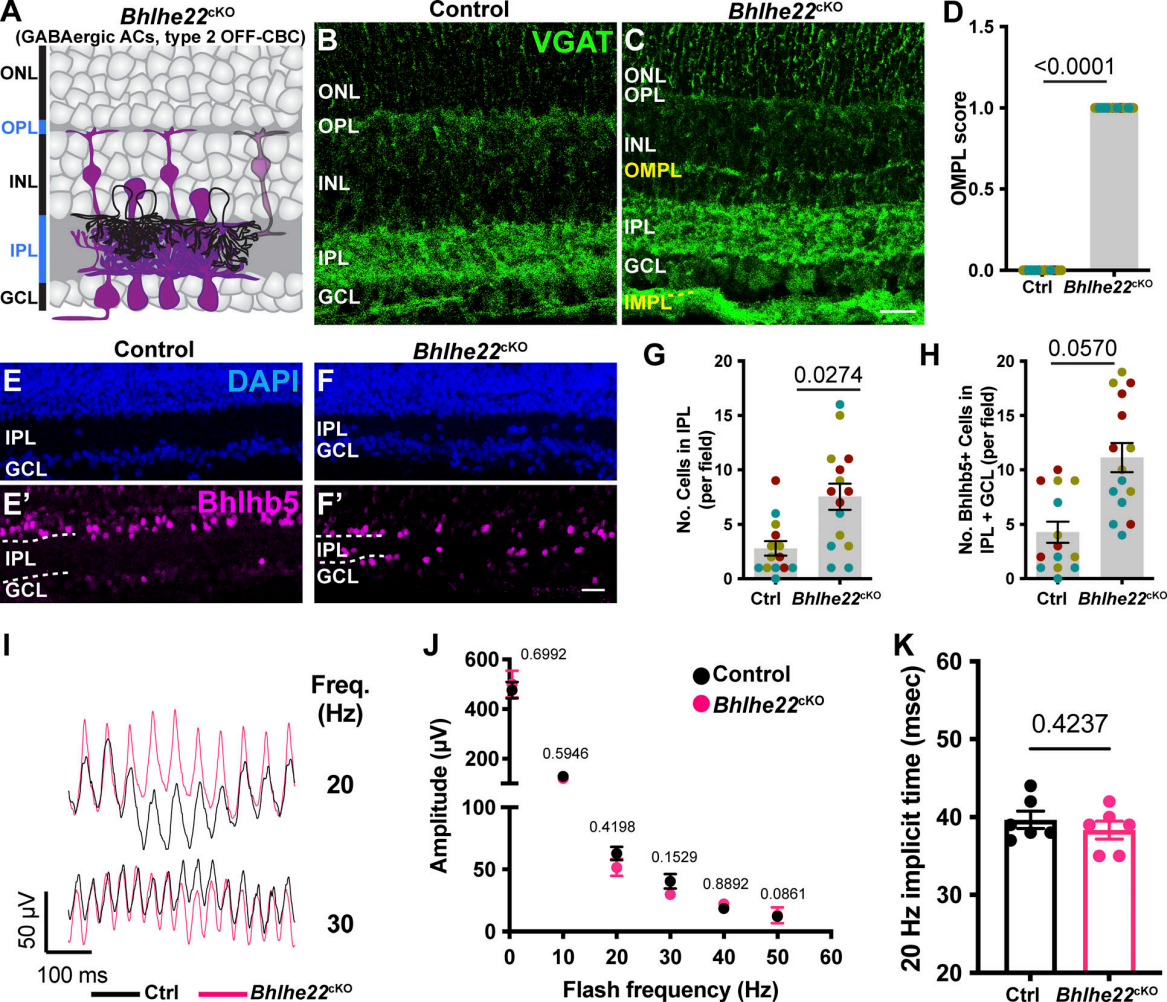
**Effect of removal of *Fat3* from OFF-CBC type 2 on flicker ERG (Related to** Fig. 4 **). (A)** Schematic representation of cells types, i.e., type 2 OFF-CBCs and GABAergic ACs, that lose *Fat3* expression in a *Bhlhe22*^CRE^ cKO mice (*Bhlhe22*^cKO^). Magenta coloring represents cell type expression of FAT3. **(B)** VGAT immunostaining for control mice (exact genotype throughout this figure: *Bhlhe22*^CRE/+^;*Fat3*^fl/+^). **(C)** VGAT immunostaining for *Bhlhe22*^cKO^ mice (exact genotype throughout this figure: *Bhlhe22*^CRE/+^;*Fat3*^fl/∆TM^). **(D)** Quantification of the OMPL score for *Bhlhe22*^cKO^. Controls: 0.0 ± 0.0 (*n* = 3 animals, 15 retinal regions); *Bhlhe22*^cKO^: 1.0 ± 0.0 (*n* = 3 animals, 18 retinal regions). Each data point corresponds to a retinal region, color-coded by animal, analyzed using a nested two-tailed test. **(E)** DAPI and Bhlhb5 (a.k.a. *Bhlhe22* gene encoded protein) staining of control mouse retinas. **(F)** DAPI and Bhlhb5 staining of *Bhlhe22*^cKO^ retinas. **(G)** Quantification of the number of nuclei per field in the IPL. Controls: 2.79 ± 0.66 (*n* = 3 animals, 14 retinal regions); *Bhlhe22*^cKO^: 7.53 ± 1.20 (*n* = 3 animals, 15 retinal regions). Each data point corresponds to a retinal region, color-coded by animal, analyzed using a nested two-tailed test. **(H)** Quantification of the number of Bhlhb5^+^ nuclei per field in the IPL and GCL. Controls: 4.29 ± 0.98 (*n* = 3 animals, 14 retinal regions); *Bhlhe22*^cKO^: 11.13 ± 1.34 (*n* = 3 animals, 15 retinal regions). Each data point corresponds to a retinal region, color-coded by animal, nested two-tailed test. **(I)** Representative flicker ERG raw traces for controls (*n* = 6 eyes) and *Bhlhe22*^cKO^ (*n* = 6 eyes) at 20 and 30 Hz. **(J)** Flicker ERG amplitude at 0.5, 10, 20, 30, 40, and 50 Hz for controls (*n* = 6 eyes) and *Bhlhe22*^cKO^ (*n* = 6 eyes). **(K)** Flicker ERG implicit time at 20 Hz for controls (39.7 ± 1.1 ms, *n* = 6 eyes) and *Bhlhe22*^cKO^ (38.3 ± 1.1 ms *n* = 6 eyes). Scale bars: 20 µm. Error bars: SEM.

We next asked how the presence of FAT3 in other retinal neurons influences retinal organization and contributes to the impaired high-frequency light response seen in *Fat3*^∆TM/∆TM^ mutant mice. To survey other FAT3^+^ cells for possible effects on the high-frequency light response, we used the *Isl1*^CRE^ mouse line to drive recombination and deletion of *Fat3* in all ON-CBCs, starburst ACs, and RGCs, but not in OFF-CBCs (Elshatory et al., 2007) (*Isl1*^cKO^, Fig. 4, A–H). Although *Isl1*^cKO^ mice exhibited all the cellular phenotypes previously described in the retina of *Fat3*^ΔTM/ΔTM^ mice (Fig. 4, B–H), the 0.5–50 Hz flicker ERG amplitude and 20 Hz implicit time were normal compared with littermate control mice (Fig. 4, I–K). Thus, the high-frequency light response defects are not due to the gross disorganization of retinal lamination and do not reflect a role for FAT3 in RGCs or ON-CBCs alone. The >30 Hz amplitude and 20 Hz implicit timing were also unaffected by removal of *Fat3* from GABAergic ACs and the type 2 OFF-CBC subset using *Bhlhe22*^CRE^ (*Bhlhe22*^cKO^ mice, Fig. S3, A–K). We were not able to directly test whether high-frequency light response requires FAT3 in OFF-CBCs, as there is no Cre line that is active in all OFF-CBCs. Nonetheless, these experiments showed that the high-frequency light response defects are not caused by retinal lamination defects and most likely involve the loss of FAT3 from OFF-CBCs.

**Figure S4.**
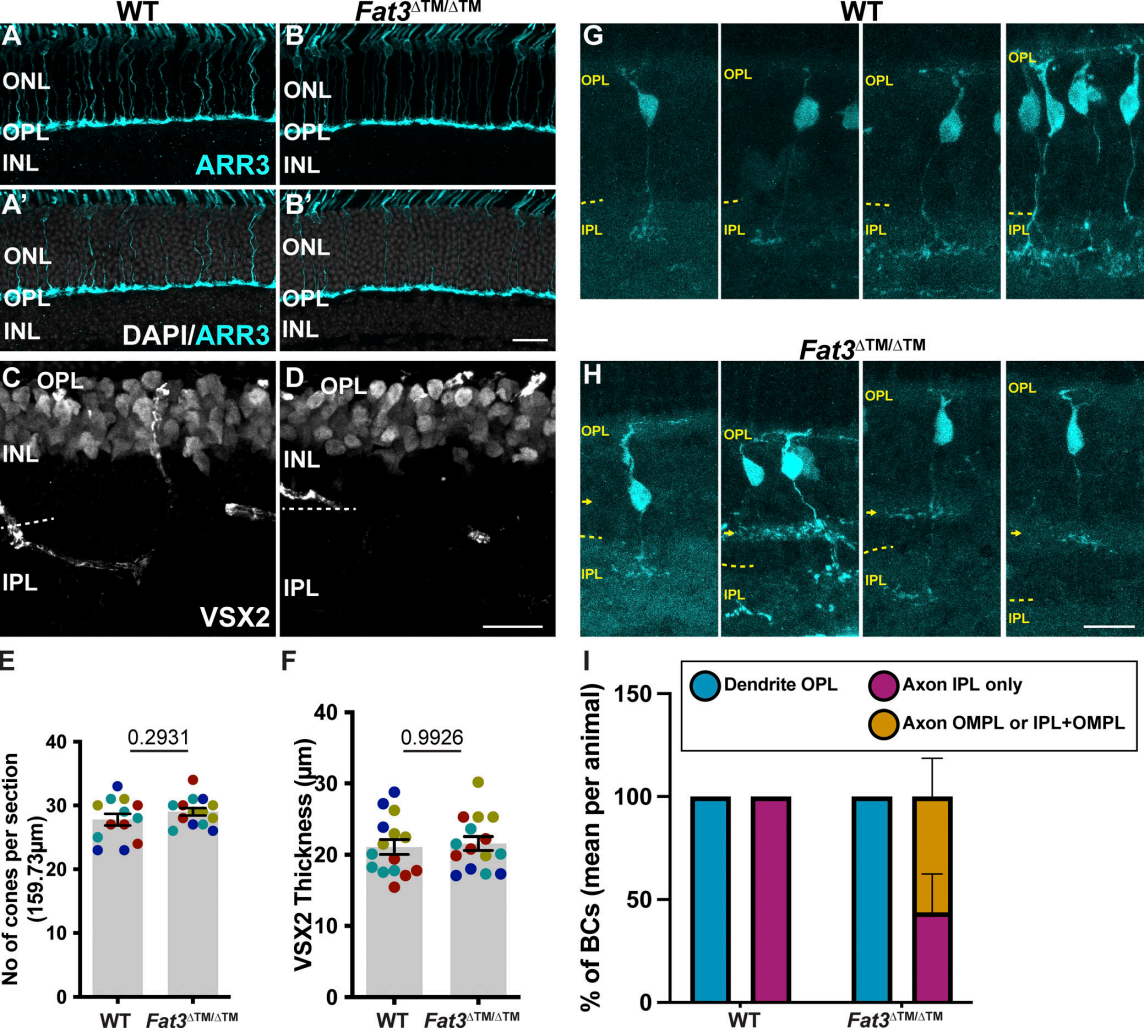
**BC and cones numbers, visualization of *Grik1*^+^ BCs morphology via injection of an AVV-Grik1-GFP virus in *Fat3* mutants (Related to** Figs. 3 and 4**). (A)** ARR3 immunohistochemistry of WT retinas. **(A′)** shows ARR3 staining together with DAPI staining. **(B)** ARR3 immunohistochemistry of *Fat3*^∆TM/∆TM^ retinas. **(B′)** shows ARR3 staining together with DAPI staining. **(C)** VSX2 immunostaining of WT retinas. **(D)** VSX2 immunostaining of *Fat3*^∆TM/∆TM^ retinas. **(E)** Quantification of number of cones marked by ARR3, as shown in A and B. WT controls: 27.77 ± 0.907 (*n* = 4 animals, 13 retinal regions); *Fat3*^∆TM/∆TM^: 29.00 ± 0.593 (*n* = 4 animals, 14 retinal regions). Each data point corresponds to a retinal region, color-coded by animal, analyzed using a nested two-tailed test. **(F)** Quantification of thickness of area occupied by VSX2 staining, as shown in C and D. WT controls: 21.09 ± 1.045 (*n* = 4 animals, 15 retinal regions); *Fat3*^∆TM/∆TM^: 21.59 ± 0.969 (*n* = 4 animals, 15 retinal regions). Each data point corresponds to a retinal region, color-coded by animal, analyzed using a nested two-tailed test. **(G)** Examples of retinal sections showing cells expressing GFP under the control of the *Grik1* enhancer. The GFP reporter was introduced through *in vivo* injection of AAV8-Grik1-GFP. WT retinas are shown in G. **(H)***Fat3*^∆TM/∆TM^ retinas injected with AAV8-Grik1-GFP. The OPL is indicated with a yellow arrowhead and the OMPL, labeled with VGAT staining, is marked with a yellow arrow. Scale bar: 20 µm. **(I)** Quantification of *Grik1*+ BC dendrites in the OPL (WT Controls: 100%; *Fat3*^∆TM/∆TM^: 100%), axons in the IPL only (WT Controls: 100%; *Fat3*^∆TM/∆TM^: 44 ± 18.6%), and axons in the OMPL or in the OMPL and IPL (WT Controls: 0%; *Fat3*^∆TM/∆TM^: 56 ± 18.6%). Controls: *n* = 5 animals, 12 cells, *Fat3*^∆TM/∆TM^: *n* = 7 animals, 22 cells. Mann–Whitney test. Error bars: SEM.

To investigate possible cellular origins of the visual deficits, we analyzed cone and BC number and morphology. We found no change in the number or distribution of cones (detected by staining for cone arrestin, ARR3) (27.77 ± 0.91 cones, *n* = 4 animals, 13 sections versus 29.00 ± 0.59 cones, *n* = 4 animals, 15 sections of WT eyes) or BCs (detected by staining for VSX2), which occupied a similar area in WT compared with *Fat3*^ΔTM/ΔTM^ mutant retinas (21.09 ± 1.04 arbitrary units, *n* = 4 animals, 15 sections versus 21.59 ± 0.97 arbitrary units, *n* = 4 animals, 15 sections from WT eyes (Fig. S4, A–F). To visualize OFF-CBC morphology, we created a novel AAV vector (AAV8-Grik1-GFP) using our previously described *Grik1* enhancer/promoter element (Kishi et al., 2019). WT and mutant P2/P3 retinas were injected with AAV8-Grik1-GFP and the position of the OFF-CBC dendrites and axon terminals was evaluated at P22. Whereas 100% (12 cells from *n* = 5 animals) of OFF-CBC axons terminated and stratified in the OFF sublaminae of the IPL in WT animals, only 44 ± 19% of the mutant OFF-CBC axons (averaged 22 cells from *n* = 7 animals) terminated in the IPL only. 56 ± 19% of *Fat3*-mutant OFF-CBC axons terminated instead in the OMPL or both the OMPL and the IPL (Fig. S4, G–I). Despite this change in axon position, mutant OFF-CBCs showed dendrites that oriented properly into the OPL, as in the WT retina (Fig. S4, G–I). Thus, unlike its role in ACs and the position of their dendrites in the IPL, FAT3 is not essential for confining OFF-CBC dendritic arbors to the OPL.

### FAT3 intracellular signaling is critical for high-frequency light ERG response and the step ERG d-wave

**Figure 5.**
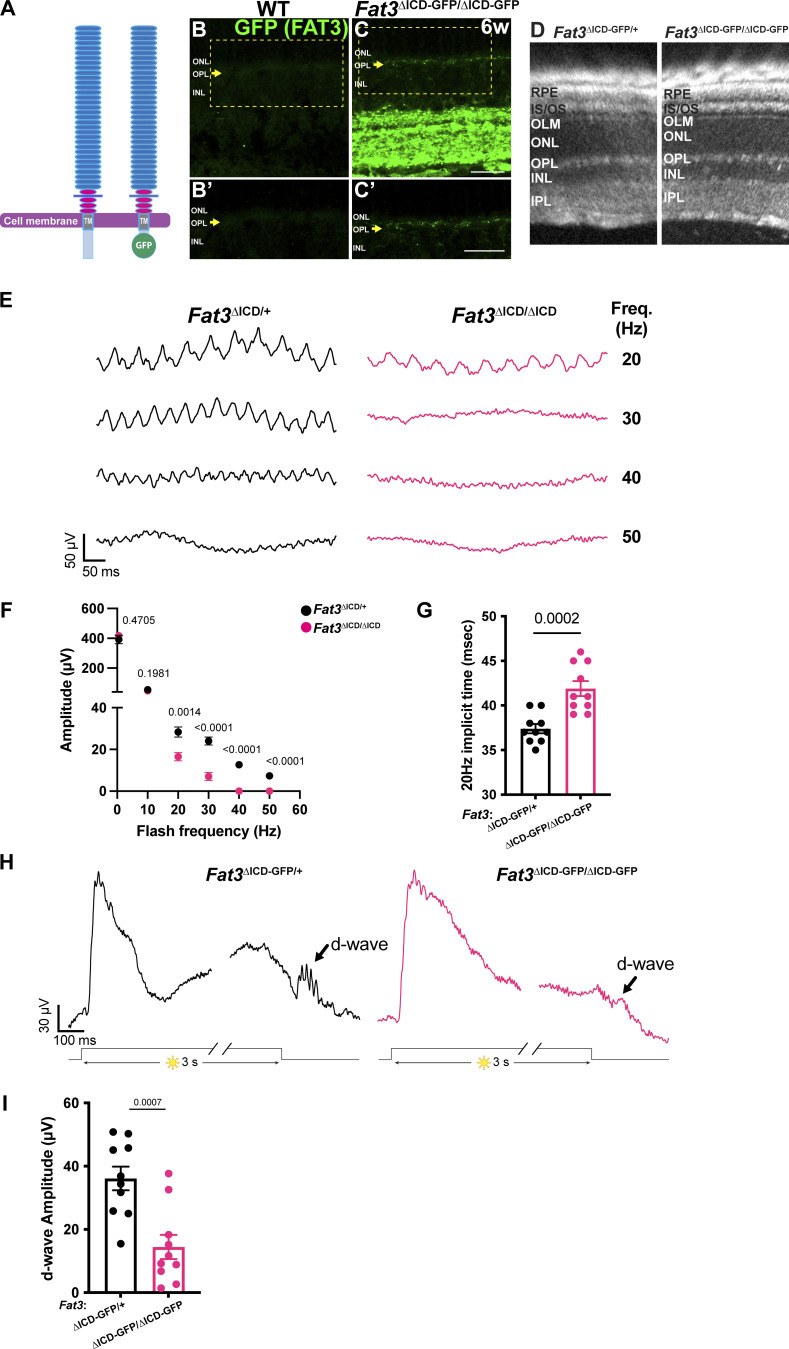
**High** **-** **frequency flicker ERG and step ERG of FAT3 ICD deficient mice. (A and B)** Schematics of molecular structure of FAT3 WT and FAT^ΔICD-GFP^ proteins B. Immunostaining for GFP in WT retinal sections. The arrow points the OPL. Inset (yellow box) is seen in B′. **(C)** Immunostaining for GFP in *Fat3*^∆ICD-GFP/∆ICD-GFP^ retinal sections. The arrow points to the OPL. Inset (yellow box) is seen in C′. **(D)** Representative OCT images of *Fat3*^ΔICD-GFP/+^ control and *Fat3*^ΔICD-GFP/ΔICD-GFP^ eyes. **(E)** Representative flicker ERG raw traces of *Fat3*^ΔICD-GFP/+^ control and *Fat3*^ΔICD-GFP/ΔICD-GFP^ eyes elicited by 3.162 cd s/m^2^ flashes at 20, 30, 40, and 50 Hz frequencies. **(F)** Flicker ERG amplitude at 0.5, 10, 20, 30, 40, and 50 Hz for *Fat3*^ΔICD-GFP/+^ control (*n* = 10 eyes) and *Fat3*^ΔICD-GFP/ΔICD-GFP^ (*n* = 10) eyes. Unpaired two-tailed Student’s *t* test. Error bar: SEM. **(G)** Flicker ERG implicit time at 20 Hz for *Fat3*^ΔICD-GFP/+^ control (37.4 ± 0.5 ms, *n* = 10 eyes) and *Fat3*^ΔICD-GFP/ΔICD-GFP^ (41.9 ± 0.8 ms, *n* = 10) eyes at 20 Hz. Unpaired two-tailed Student’s *t* test. Error bar: SEM. **(H)** Representative step ERG raw traces of *Fat3*^ΔICD/+^ control (left) and *Fat3*^ΔICD-GFP/ΔICD-GFP^ (right) eyes elicited by a 3-s step light at 1,000 cd/m^2^ intensity. **(I)** Quantification of step ERG d-wave amplitudes for *Fat3*^ΔICD-GFP/+^ control (36.1 ± 3.7 µV, *n* = 10 eyes) and *Fat3*^ΔICD-GFP/ΔICD-GFP^ (14.4 ± 3.8 µV, *n* = 10) eyes elicited by a 3-s step of light at 1,000 cd/m^2^ intensity. Unpaired two-tailed Student’s *t* test. Error bar: SEM.

FAT3 is a versatile protein that can mediate non-autonomous interactions *via* its extracellular domain and autonomous effects by recruiting different sets of cytoskeletal effectors to its ICD (Avilés et al., 2022). To better understand how FAT3 signaling supports high frequency visual signal transmission and contributes to the step ERG d-wave, we analyzed *Fat3*^∆ICD-GFP^ animals, in which most of the ICD is replaced with GFP while keeping the extracellular and transmembrane domains anchored to the cell membrane (Avilés et al., 2022) (Fig. 5 A). These animals showed expression of the FAT3-GFP fusion protein in the OPL (Fig. 5, B and C), consistent with FAT3 protein localization (Fig. 3 D). In *Fat3*^∆ICD-GFP/∆ICD-GFP^ mutant mice, ACs migrate to abnormal cell layers and fail to retract their neurites, but do not form ectopic plexiform layers, as shown previously (Avilés et al., 2022). Therefore, analyzing *Fat3*^∆ICD-GFP/∆ICD-GFP^ mutants can reveal whether high-frequency light response depends on intracellular signaling and/or the ability to form ectopic plexiform layers.

Consistent with previous histological analysis (Avilés et al., 2022), no ectopic plexiform layers were detected by OCT *in vivo* imaging of *Fat3*^ΔICD-GFP/ΔICD-GFP^ eyes (Fig. 5 D). Nonetheless, the amplitude of the >20 Hz flicker ERG response was decreased in *Fat3*^ΔICD-GFP/ΔICD-GFP^ mice, as in *Fat3*^ΔTM/ΔTM^ mice (Fig. 5, E and F). Likewise, the 20-Hz implicit time was delayed and the amplitude of the d-wave in step ERG responses was decreased (Fig. 5, G–I). These changes in *Fat3*^ΔICD-GFP/ΔICD-GFP^ mice are highly similar to those in *Fat3*^ΔTM/ΔTM^ mice (Fig. 1, E, F, and I). The loss of high-frequency flicker ERG responses even in the absence of ectopic plexiform layers further underscores that changes in AC wiring do not contribute to altered visual function in *Fat3* mutant mice. Rather, these results suggest a potential role for FAT3 signaling in OFF-CBCs, in keeping with the results of the genetic analyses described above.

### GRIK1 localization to the synapse is reduced in *Fat3* mutants

**Figure 6.**
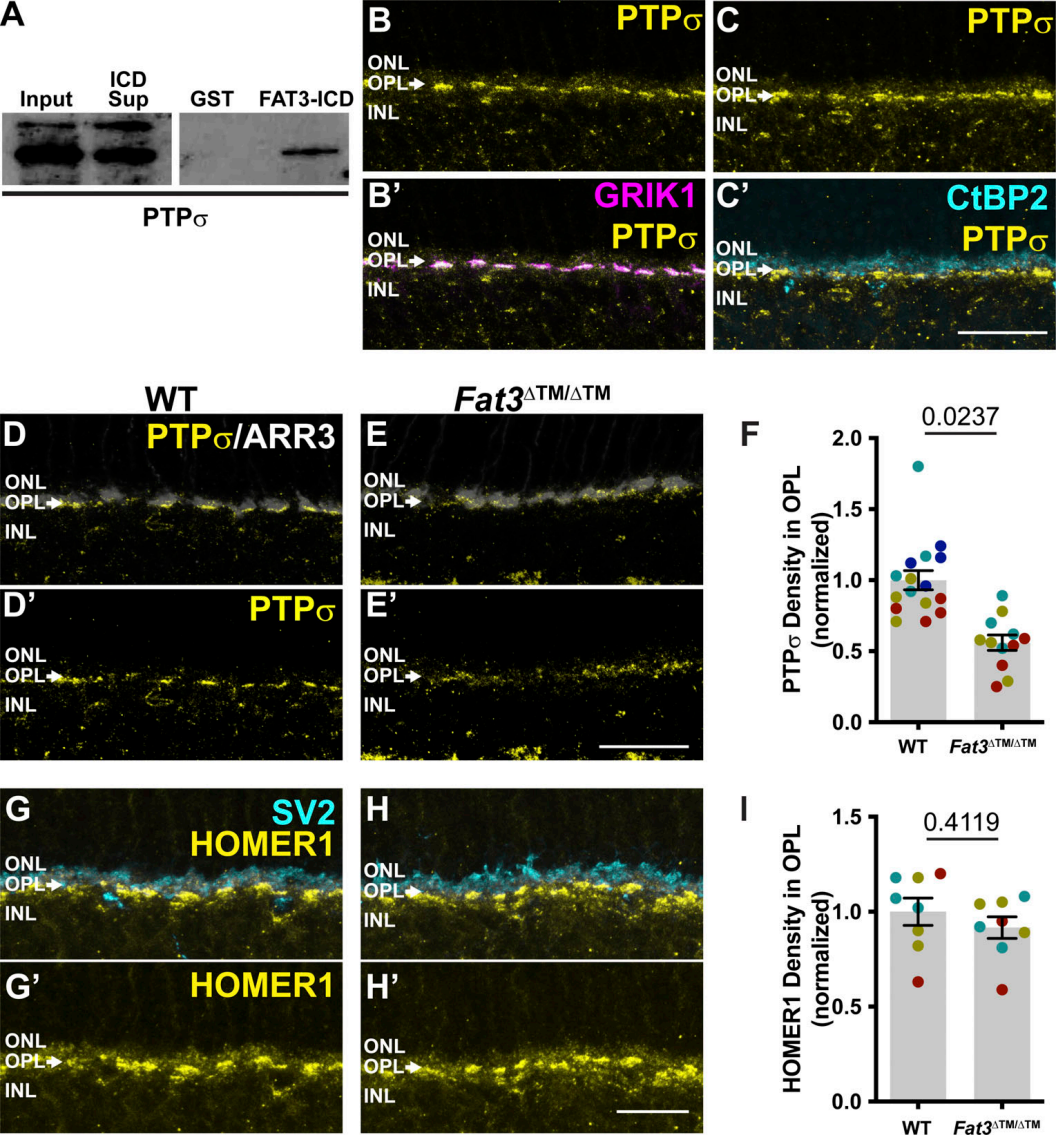
**PTPσ and HOMER1 localization in WT and FAT3 mutant retinas. (A)** Western blot for PTPσ in protein lysate (input), in the supernatant after pulldown with a GST fusion to FAT-ICD (sup), and in the pellet after pulldown with GST alone (GST) or a FAT3-ICD GST fusion protein. **(B and B′)** Immunostaining of PTPσ (yellow) and GRIK1 (magenta, B′) in the OPL region of WT retinal sections. **(C and C″)** Immunostaining of PTPσ (yellow) and CtBP2 (cyan, C″), a marker of presynaptic terminals in photoreceptor axons. **(D and D′)** Immunostaining of PTPσ (yellow) and ARR3 (white) and PTPσ (D′) alone in the OPL region of WT retinal sections. **(E and E′)** Immunostaining of PTPσ (yellow) and ARR3 (white) and PTPσ (E′) alone in the OPL region of *Fat3*^∆TM/∆TM^ retinal sections. **(F)** Quantification of PTPσ integrated intensity (normalized) in the OPL. WT Controls: 1.00 ± 0.07 (*n* = 4 animals, 16 retinal regions); *Fat3*^∆TM/∆TM^: 0.56 ± 0.05 (*n* = 3 animals, 12 retinal regions). Each data point corresponds to a retinal region, color-coded by animal, nested two-tailed test. Error bar: SEM. **(G and G′)** Immunostaining of HOMER1 (yellow) and SV2 (cyan), and HOMER1 (G′) alone in WT retinal sections. **(H and H′)** Immunostaining of HOMER1 (yellow) and SV2 (cyan), and HOMER1 (H′) alone in *Fat3*^∆TM/∆TM^ retinal sections. **(I)** Quantification of HOMER1 integrated intensity in the OPL. WT Controls: 1.00 ± 0.07 (*n* = 3 animals, eight retinal regions); *Fat3*^∆TM/∆TM^: 0.91 ± 0.06 (*n* = 3 animals, eight retinal regions). Each data point corresponds to a retinal region, color-coded by animal, nested two-tailed test. Error bar: SEM. Scale bars: 20 µm. Source data are available for this figure: SourceData F6.

**Figure S5.**
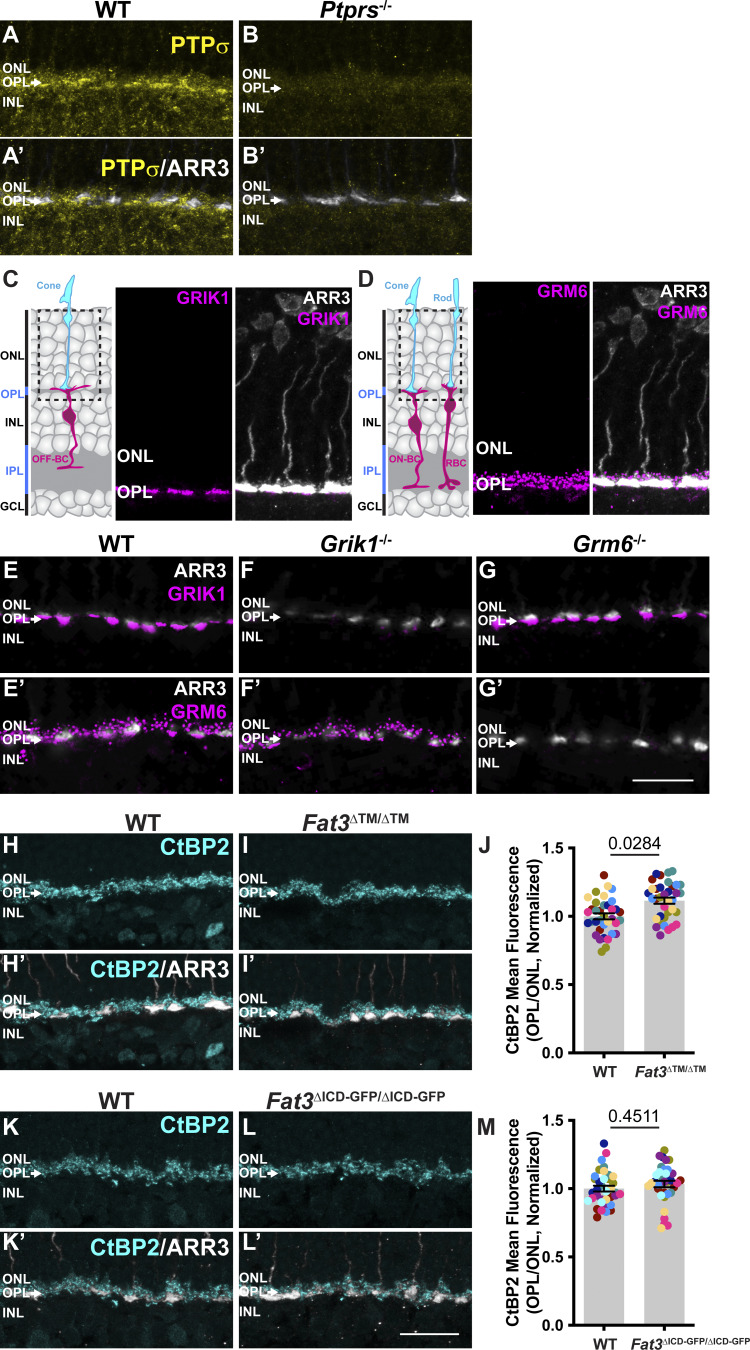
**Expression pattern of postsynaptic components PTPσ, HOMER1, GRIK1, and GRM6 in the retina (Related to** Figs. 6 and 7**). (A and B)** PTPσ immunostaining in WT retinas B. PTPσ immunostaining in *Ptprs*^−/−^ retinas. **(A′ and B′)** Cone arrestin (ARR3) labels the cone photoreceptors endings in the OPL in A′ and B′. **(C)** Schematic representation of OFF-BCs and their synapses with cone photoreceptors. ARR3 (white) labels cones and GRIK1 (magenta) labels postsynaptic BC dendrites. **(D)** Schematic representation of ON-BCs and their synapses with cone photoreceptors. ARR3 (white) labels cones and GRM6 (magenta) labels postsynaptic cone and rod BC (RBC) dendrites. **(E and E′)** GRIK1 and GRM6 (E′) immunostaining of adult WT retina. **(F and F′)** GRIK1 and GRM6 (F′) immunostaining of *Grik1*^−/−^ retina. **(G and G′)** GRIK1 and GRM6 (G′) immunostaining of *Grm6*^−/−^ retina. **(H)** Immunostaining for CtBP2, a marker of presynaptic ribbon in WT retinas. **(I)** Immunostaining for CtBP2 *Fat3*^∆TM/∆TM^ retinas. **(H′ and I′)** Cone arrestin (ARR3) labels the cone photoreceptors endings in the OPL in H′ and I′. **(J)** Quantification of CtBP2 mean fluorescence intensity in the OPL, normalized by ONL signal. WT controls: 1.00 ± 0.02 (*n* = 8 animals, 36 retinal regions); *Fat3*^∆TM/∆TM^: 1.11 ± 0.02 (*n* = 8 animals, 36 retinal regions). Each data point corresponds to a retinal region, color-coded by animal, analyzed using a nested two-tailed test. **(K)** Immunostaining for CtBP2 in WT retinas, littermates of *Fat3*^∆ICD-GFP/∆ICD-GFP^ animals. **(L)** Immunostaining for CtBP2 in *Fat3*^∆ICD-GFP/∆ICD-GFP^ retinas. **(K′ and L′)** Cone arrestin (ARR3) labels the cone photoreceptors endings in the OPL in K′ and L′. **(M)** Quantification of CtBP2 mean fluorescence intensity in the OPL, normalized by ONL signal. WT controls: 1.00 ± 0.02 (*n* = 9 animals, from 36 retinal regions); *Fat3*^∆ICD/∆ICD^: 1.03 ± 0.02 (*n* = 9 animals, from 36 retinal regions). Each data point corresponds to a retinal region, color-coded by animal, analyzed using a nested two-tailed test. Scale bars: 20 µm. Error bars: SEM.

Given the absence of obvious changes in the position or morphology of BC dendrites (Fig. S4), we hypothesized that the observed changes in retinal function are due instead to FAT3-dependent effects on retinal synapses. Indeed, in addition to binding to a variety of cytoplasmic effectors important for neuronal migration and neurite retraction, GST-pulldown assays showed that the FAT3 ICD binds to several proteins associated with synaptic function, such as HOMER1 (Avilés et al., 2022) and the LAR family protein, PTPσ, which is encoded by the *Protein tyrosine phosphatase receptor type S* (*Ptprs*) gene (Fig. 6 A). Further, *Drosophila* Fat-like interacts with the related receptor-type protein tyrosine phosphatase (RPTP) protein dLAR (Barlan et al., 2017), and PTPσ is required for excitatory synapse formation in hippocampal neurons (Han et al., 2018; Horn et al., 2012; Roppongi et al., 2020). Since *Ptprs* RNA is detected in both ON- and OFF-BCs (Shekhar et al., 2016) (Fig. S2), we asked whether cell type-specific synaptic features might be altered in the absence of FAT3. Immunostaining revealed that PTPσ localized to the postsynaptic dendrites of CBCs, overlapping with the postsynaptic glutamate receptor subunit GRIK1 (Fig. 6 B and Fig. S5, A–G) and apposing CtBP2 in the ARR3^+^ cone terminals (Fig. 6 C). However, significantly less PTPσ was detected in the OPL of *Fat3*^ΔTM/ΔTM^ mutants (density in the OPL of 0.56 ± 0.05, *n* = 3 mutant animals versus 1.00 ± 0.07 in *n* = 4 WT animals) (Fig. 6, D–F). Immunostaining revealed no change in the levels of postsynaptic HOMER1, which continued to oppose SV2^+^ synaptic terminals in the OPL of *Fat3* mutants (Fig. 6, G–I). Presynaptic CTBP2^+^ ribbons were also not decreased in *Fat3* mutants (OPL normalized mean fluorescence intensity: 1.11 ± 0.02 in *n* = 8 *Fat3*^ΔTM/ΔTM^ animals versus 1.00 ± 0.02 in *n* = 8 WT animals; 1.03 ± 0.02 in *n* = 9 *Fat3*^ΔICD-GFP/ΔICD-GFP^ animals versus 1.00 ± 0.02 in *n* = 9 WT animals) (Fig. S5, H–M), suggesting that FAT3 may not be required for synapse formation *per se*.

**Figure 7.**
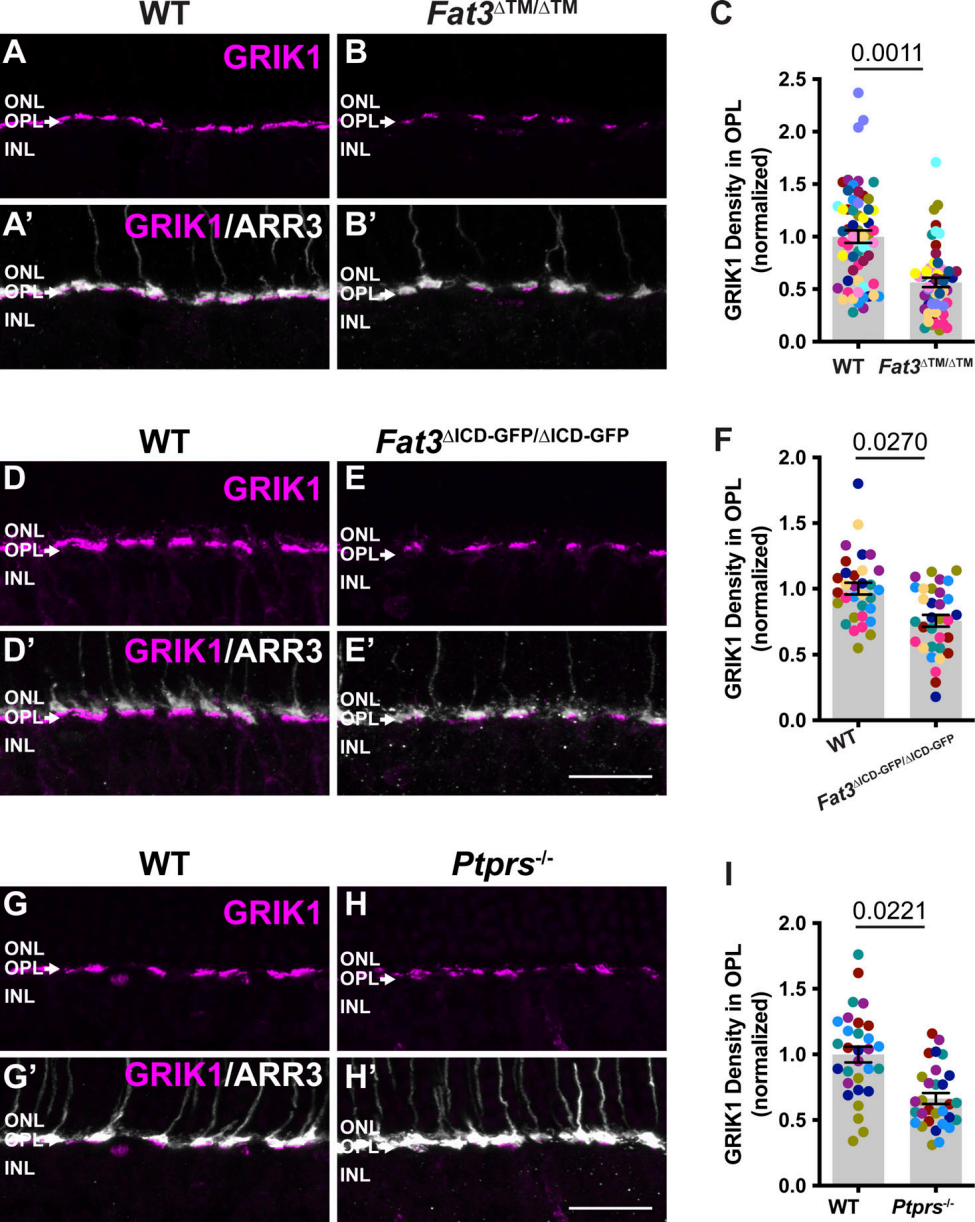
**GRIK1 localization in WT, FAT3, and PTPσ mutant retinas. (A)** Immunostaining for GRIK1 (magenta) in the OPL region of WT retinal sections. **(B)** Immunostaining for GRIK1 (magenta) in *Fat3*^∆TM/∆TM^ retinal sections. **(A′ and B′)** Cone arrestin (ARR3) (white) labels the cone photoreceptor axonal endings in the OPL in A′ and B′. **(C)** Quantification of GRIK1 integrated intensity in the OPL. WT Controls: 1.00 ± 0.06 (*n* = 13 animals, 60 retinal regions); *Fat3*^∆TM/∆TM^: 0.56 ± 0.04 (*n* = 12 animals, 54 retinal regions). Each data point corresponds to a retinal region, color-coded by animal, nested two-tailed test. Error bar: SEM. **(D)** Immunostaining for GRIK1 (magenta) in the OPL region of WT retinal sections. **(E)** Immunostaining for GRIK1 (magenta) in the OPL region of *Fat3*^∆ICD-GFP/∆ICD-GFP^ retinal sections. **(D′ and E′)** Cone arrestin (ARR3) (white) labels the cone photoreceptor endings in the OPL in D′ and E′. **(F)** Quantification of GRIK1 integrated intensity in the OPL. WT Controls: 1.00 ± 0.04 (*n* = 8 animals, 32 retinal regions); *Fat3*^∆ICD/∆ICD^: 0.75 ± 0.04 (*n* = 8 animals, 32 retinal regions). Each data point corresponds to a retinal region, color-coded by animal, nested two-tailed test. Error bar: SEM. **(G)** Immunostaining for GRIK1 (magenta) in the OPL region of WT retinal sections. **(H)** Immunostaining for GRIK1 (magenta) in *Ptprs*^−/−^ retinal sections. **(G′ and H′)** Cone arrestin (ARR3) (white) labels the cone photoreceptor endings in the OPL in G′ and H′. **(I)** Quantification of GRIK1 integrated intensity in the OPL. WT Controls: 1.00 ± 0.06 (*n* = 6 animals, 30 retinal regions); *Ptprs*^−/−^: 0.66 ± 0.04 (*n* = 6 animals, 30 retinal regions). Each data point corresponds to a retinal region, color-coded by animal, nested two-tailed test. Error bar: SEM. Scale bars: 20 µm.

**Figure S6.**
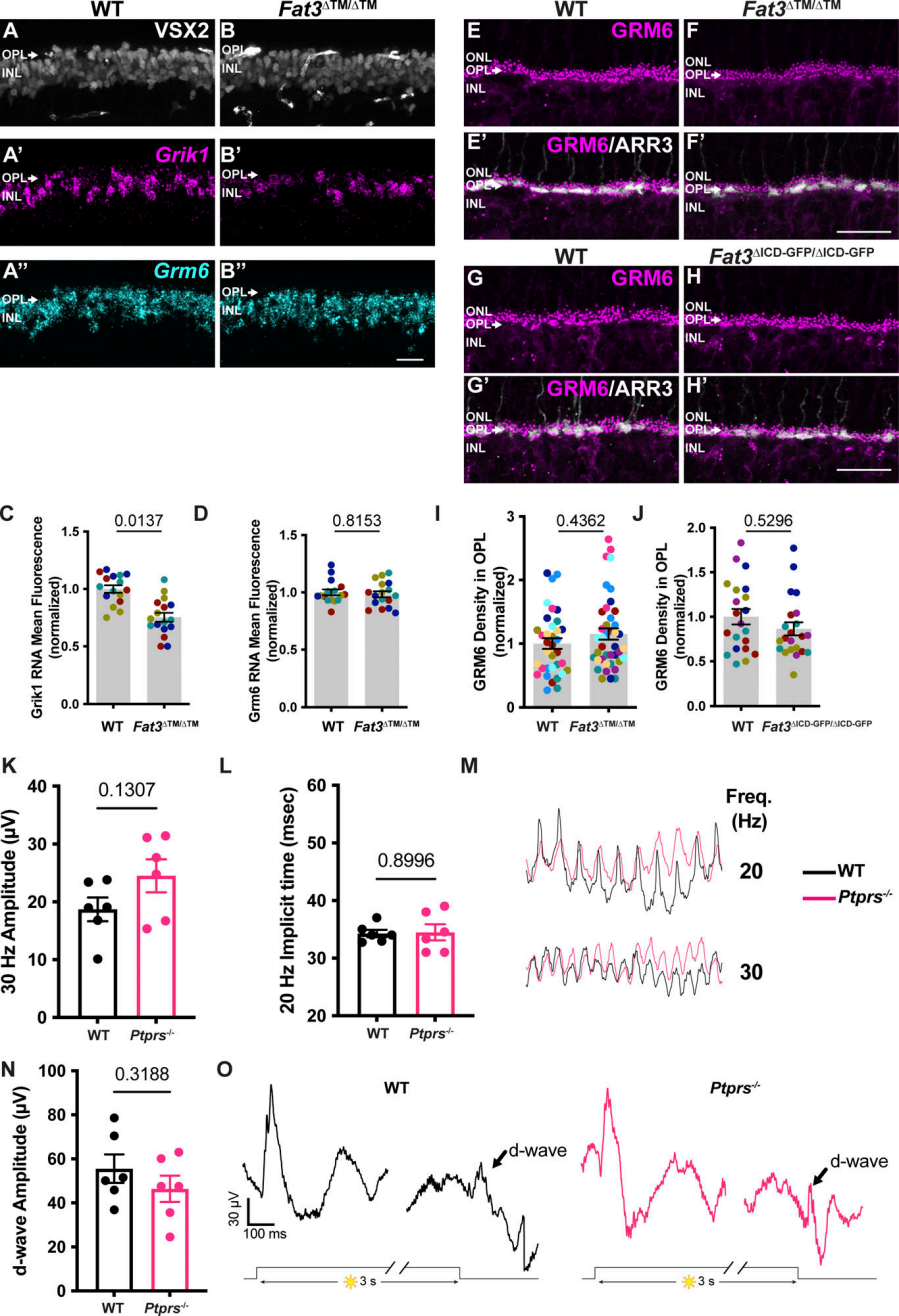
***In situ* hybridization of *Grik1* and *Grm6* and immunostaining of GRM6 upon loss of *Fat3* in mouse retina (Related to** Fig. 7 **). (A–A″)** VSX2 immunostaining (A) after *in situ* hybridization for *Grik1* (A′) and *Grm6* (A″) in WT retina. **(B–B″)** VSX2 immunostaining (B) after *in situ* hybridization for *Grik1* (B′) and *Grm6* (B″) in *Fat3*^∆TM/∆TM^ retina. **(C)** Quantification of *Grik1* RNA mean fluorescence intensity in bipolar cells. WT: 1.00 ± 0.03, *n* = 4 animals, 16 retinal regions; *Fat3*^∆TM/∆TM^: 0.75 ± 0.04, *n* = 4 animals, 17 retinal regions. Each data point corresponds to a retinal region, color-coded by animal, analyzed using a nested two-tailed test. **(D)** Quantification of *Grm6* RNA mean fluorescence intensity in bipolar cells. WT: 1.00 ± 0.02, *n* = 4 animals, from 16 retinal regions; *Fat3*^∆TM/∆TM^: 0.98 ± 0.02, *n* = 4 animals, from 17 retinal regions. Each data point corresponds to a retinal region, color-coded by animal, analyzed using a nested two-tailed test. **(E)** Immunostaining for GRM6 in WT retinas. **(F)** Immunostaining for GRM6 in *Fat3*^∆TM/∆TM^ retinas. **(E′ and F′)** Cone arrestin (ARR3) labels the cone photoreceptors endings in the OPL in E′ and F′. **(G)** Immunostaining for GRM6 in WT retinas, littermates of *Fat3*^∆ICD-GFP/∆ICD-GFP^ animals. **(H)** Immunostaining for GRM6 in *Fat3*^∆ICD-GFP/∆ICD-GFP^ retinas. Cone arrestin (ARR3) labels the cone photoreceptors endings in the OPL in G′ and H′. **(I)** Quantification of GRM6 integrated intensity in the OPL. WT controls: 1.00 ± 0.08 (*n* = 8 animals, 35 retinal regions); *Fat3*^∆TM/∆TM^: 1.15 ± 0.09 (*n* = 9 animals, 41 retinal regions). Each data point corresponds to a retinal region, color-coded by animal, nested two-tailed test. **(J)** Quantification of GRM6 integrated intensity in the OPL. WT Controls: 1.00 ± 0.09 (*n* = 5 animals, 21 retinal regions); *Fat3*^∆ICD/∆ICD^: 0.86 ± 0.07 (*n* = 5 animals, 22 retinal regions). Each data point corresponds to a retinal region, color-coded by animal, analyzed using a nested two-tailed test. **(K)** Flicker ERG amplitude at 30 Hz for WT (18.70 ± 2.05 µV *n* = 6 eyes) and *Ptprs*^−/−^ retinas (24.50 ± 2.86 µV, *n* = 6 eyes). **(L)** Flicker ERG implicit time at 20 Hz for WT (34.3 ± 0.6 ms, *n* = 6 eyes) and *Ptprs*^−/−^ retinas (34.5 ± 1.4 ms, *n* = 6 eyes). **(M)** Representative flicker ERG raw traces of WT control and *Ptprs*^−/−^ eyes elicited by 3.162 cd s/m^2^ flashes at 20 and 30 Hz frequencies. **(N)** Quantification of step ERG d-wave amplitudes of WT (55.58 ± 6.43 µV *n* = 6 eyes) and *Ptprs*^−/−^ (46.40 ± 5.94 µV *n* = 6 eyes) elicited by a 3-s step of light at 1,000 cd/m^2^ intensity. **(O)** Representative step ERG raw traces of WT (*n* = 6) control and *Ptprs*^−/−^ (*n* = 6) eyes elicited by a 3-s step light at 1,000 cd/m^2^ intensity. Scale bars: 20 µm. Error bars: SEM.

GRIK1 is the only subunit of ionotropic glutamate receptors (iGluR) enriched specifically in OFF-CBCs (Amamoto et al., 2019; Kishi et al., 2019; Shekhar et al., 2016; West et al., 2022) (Fig. S5, C, E, and F). GRIK1 staining was strongly reduced in the OPL of both *Fat3*^ΔTM/ΔTM^ (Fig. 7, A–C) and *Fat3*^ΔICD-GFP/ΔICD-GFP^ (Fig. 7, D–F) retinas compared with WT littermates (normalized density in the OPL: 0.56 ± 0.04, *n* = 12 *Fat3*^ΔTM/ΔTM^ versus 1.00 ± 0.06 in *n* = 13 WT animals, and 0.75 ± 0.04 in *n* = 8 *Fat3*^ΔICD-GFP/ΔICD-GFP^ versus 1.00 ± 0.04 in *n* = 8 WT animals). BCs also expressed less *Grik1* RNA in *Fat3*^ΔTM/ΔTM^ animals compared with WT (Fig. S6, A–C). By contrast, the ON-CBC synaptic protein GRM6, which is a metabotropic glutamate receptor subunit (Fig. S5, D and G), was present at similar levels in the OPL of mutant and control retinas (Fig. S6, E, F, and I) and in *Fat3*^ΔICD-GFP/ΔICD-GFP^ mutants (Fig. S6, G, H, and J). There was also no difference in the level of *Grm6* RNA in the INL of mutant and control retinas (Fig. S6, A, B, and D). Although the loss of *Ptprs* also resulted in a decrease of GRIK1 in the OPL (Fig. 7, G–I), *Ptprs*^−/−^ animals did not show any change in the ERG response to a 30-Hz flickering stimulus, the implicit timing of the response to a 20-Hz stimulus or the d-wave amplitude in the step ERG assay (Fig. S6, K–O). Thus, FAT3 likely impacts not only the presence of PTPσ and GRIK1 but also additional features of the CBC synapses necessary for responses to high temporal frequency stimuli.

## Discussion

A major goal in retinal physiology is to understand how the diverse cell types of the retina, including >80 types of interneurons, properly connect to form and operate within parallel circuits that transform information about specific elements of the visual world. A valuable approach is to characterize retinal physiology and visual behavior in mice carrying mutations in genes required for proper connectivity and/or function. However, the nature of the retinal neurons that enable responses to stimuli that change with high-temporal frequency has been explored very little, in part, because standard assays of retinal function, particularly the conventional scotopic and photopic ERGs, are not designed to detect signals elicited by these stimuli. Despite lacking the cellular level resolution that can be achieved by *ex vivo* patch clamping, the ERG is a relatively simple measurement that can be used *in vivo* to reveal behaviorally relevant changes in overall retinal function from populations of cells. Here, we used flicker and step ERGs to show that retinal activity in response to high-frequency flickering and light-off stimuli is altered in *Fat3* mutant mice, which also show an impaired behavioral response to flickering stimuli. FAT3 is a multifunctional transmembrane protein that is expressed by ACs, BCs, and RGCs and is required for proper lamination of retinal neurons and their synapses. By analyzing the consequences of *Fat3* deletion from different retinal cell types, we discovered that the loss of high-frequency light response was not due to changes in AC position or connectivity. Instead, these visual defects appear to be due to abnormal BCs. Although the precise nature of the synaptic defects remains unclear, we found that FAT3 binds to the synaptic protein PTPσ and that both FAT3 and PTPσ are required for enrichment of GRIK1 at the cone to OFF-CBC synapse. Together, these data uncover a role for FAT3 in the formation and/or maintenance of cone-to-BC synapses and for retinal responses associated with signaling through these synapses.

### FAT3 mutant mice show deficits in high-frequency light ERG responses

Despite their severe defects in neuronal wiring, *Fat3* mutant mice are not blind, as seen by the preservation of conventional ERG and optomotor responses. However, when presented with fast flickering stimuli, *Fat3* mice showed severe visual deficits consistent with defects in synaptic transmission from cones to OFF-CBCs. In support of this idea, abnormal high temporal frequency light response was observed only in mice lacking *Fat3* in BCs and did not correlate with effects on retinal lamination (Figs. 1, 2, and 4). Additionally, *Fat3* is expressed by multiple BC types, including GRIK1-positive OFF-CBCs, and GRIK1 levels were reduced in the OPL of *Fat3* mutant mice (Figs. S2, 3, and 7), as were levels of PTPσ, a synaptic protein that binds to FAT3 (Fig. 6). To independently assay OFF-CBC response to light, we established a simple and non-invasive step ERG protocol and showed that the d-wave was reduced in *Fat3* mutant mice compared with WT littermates (Fig. 1). Collectively, these data suggest that FAT3 is required for proper OFF-CBC function and the ability to detect fast flickering stimuli. This phenotype is fundamentally different from that which occurs in *Fat3* mutant ACs, which extend extra neurites that form ectopic synapses.

BCs are the first retinal interneurons that encode, segregate, and relay visual information into over a dozen pathways for further processing. It had been hypothesized that the OFF-CBCs mediate high-frequency visual signal transmission, based upon indirect evidence from flicker ERG studies of mice carrying mutations such as *Grm6*^−/−^ (Tanimoto et al., 2015). OFF-CBC activities are thought to be initiated by two classes of ionotropic GluRs, namely kainate and AMPA receptors, which were proposed to differentially encode temporal signals from cones (DeVries, 2000). AMPA receptors also have been reported to mediate high-frequency signaling in cb2, a particular OFF-CBC subtype in ground squirrel retina, as shown by *ex vivo* patch recording (Grabner et al., 2016). However, due to the lack of reliable antibodies, we were not able to investigate *Fat3* mutant mice for changes in AMPA receptor level or localization.

It is unclear whether the observed changes in ERG responses are entirely due to altered OFF-CBC activity. In addition to OFF-CBCs, *Fat3* RNA is weakly expressed in the 5D and 6 ON-CBC subtypes (Shekhar et al., 2016) (Fig. S2), possibly affecting some ON-CBC functions directly. However, it is unlikely that a cell-autonomous defect in only two subtypes would alter the overall ON-CBC population responses that the ERG measures. Further, *Fat3* mutants showed no detectable change in the level of GRM6, the glutamate receptor that leads to membrane voltage changes in ON-CBCs (Masu et al., 1995). In addition, non-cell-autonomous effects are possible, as OFF-CBCs are connected to ON-BCs via AII ACs in the IPL. Thus, modulation originating within the IPL could affect OPL synaptic transmission through a backpropagation mechanism, which has not been described but was suggested by an *ex vivo* study using pharmacological blockers within the amphibian retina (Awatramani et al., 2001). For example, PTPσ might serve as an intermediary, as it can act in *trans*, is expressed in both ON and OFF-BCs (Shekhar et al., 2016) (Fig. S2), and is reduced in *Fat3* mutants (Fig. 6). Further studies, including detailed patch clamp analysis, are needed to address these possibilities and to pinpoint which defects in CBC function contribute to the flicker altered ERG response observed in FAT3 mutants.

### *Fat3* effects on high temporal frequency light ERG responses requires intracellular signaling

The discovery of a defect in synapses within the OPL of *Fat3* mutant retinas highlights FAT3’s versatility as a signaling molecule. Previous work showed that FAT3 acts through different motifs in its ICD to control AC migration, neurite retraction, and synapse localization, likely by recruiting different combinations of cytoplasmic effectors (Avilés et al., 2022). Here, we found that the FAT3 ICD is also required for the proper organization of the synapses between cones and OFF-CBCs, possibly acting through a separate module of synapse-related effectors. The FAT3-ICD interacts with several known synaptic proteins, including PTPσ, which is one of the four type IIA family of RPTP in the LAR-RPTP subfamily (Cornejo et al., 2021). Interactions between FAT3 and PTPσ appear to regulate the amount of GRIK1 at the synapse since PTPσ levels were reduced in the OPL of *Fat3* mutants (Fig. 6) and GRIK1 levels were reduced in the OPL of both *Fat3* and *Ptprs* mutants (Fig. 7). Although PTPσ plays a well-established role in the differentiation of the presynaptic component of excitatory synapses in the brain (Cornejo et al., 2021; Han et al., 2018; Roppongi et al., 2020), our findings point to a role on the postsynaptic side, as suggested previously (Horn et al., 2012). Further, the related protein LAR also can be localized to the postsynaptic compartment and is required for proper surface expression and clustering of AMPA receptors (Wyszynski et al., 2002). Thus, it is possible that PTPσ acts similarly to control the distribution of GRIK1 in CBCs, either on its own or in collaboration with FAT3. Since *Ptprs* mutants do not show the same visual deficits as *Fat3* mutants (Fig. S6), other LAR subfamily members may compensate. This might also explain why *Ptprs* mutants have no obvious changes in retinal lamination (Sapieha et al., 2005). Alternatively, other FAT3-dependent proteins may enable sufficient GRIK1 activity for OFF-CBC responses in *Ptprs* mutants. Although much remains to be learned about the contribution of FAT3-PTPσ interactions to the synapse, this seems to be a conserved relationship since *Drosophila* Fat-like and LAR interact to ensure collective cell migration (Barlan et al., 2017), in this case acting in *trans* (Williams and Horne-Badovinac, 2023, *Preprint*). It also remains unclear whether loss of FAT3 also affects any features of the AC synapses, either those made in ectopic plexiform layers, or those properly positioned in the IPL.

There are several ways that FAT3 might influence synaptic function. One model is that the FAT3 ICD serves as a scaffold for synaptic proteins that secures them to the OFF-CBC dendrites in the OPL and thus directly shapes visual signal transmission. This fits with the fact that FAT3, PTPσ, and GRIK1 are all localized to the OPL and that several other synaptic proteins, including the WAVE regulatory complex, also interact with the FAT3 ICD (Avilés et al., 2022). Alternatively, FAT3 may ensure directed trafficking of PTPσ and other proteins to the synapse, echoing its role as a tissue polarity protein and its ability to promote asymmetric localization of cytoskeletal proteins (Krol et al., 2016). These possibilities are not mutually exclusive as FAT3 could control synapse assembly during development and then maintain the synapse in the mature retina. Finally, FAT3 may impact the synapse through effects on gene expression. In flies, the Fat-like ICD is cleaved and binds to a transcriptional co-repressor to influence gene expression (Fanto et al., 2003). Since the LAR ICD can be internalized and thus inhibit transcription (Haapasalo et al., 2007), FAT3 and PTPσ could cooperate to control the expression of synaptic genes. This notion is supported by the observation that *Grik1* RNA is reduced in *Fat3* mutant BCs (Fig. S6). Regardless of how FAT3 acts, additional work is needed to understand how changes in the molecular composition of cone-CBC synapses impair synaptic function either at the single-cell level or at the circuit level, where non-cell-autonomous effects on cones or other CBCs may contribute.

### Limitations of the study

Although our data provide strong evidence that FAT3 impacts the OFF-CBC signaling needed for high-frequency light response, this study does not show definitively what is wrong at the level of the synapse. The ERG is a measurement of cellular activity across a population of cells, i.e., a group of cells from the same class that functions similarly, such as OFF-CBC versus ON-CBC. ERGs are not designed to detect signals carried within distinct microcircuits that perform transformations of particular features of a visual scene. Additionally, due to the lack of Cre lines specific for all BCs, or only OFF-CBCs or ON-CBCs, it is not possible to analyze the consequences of FAT3 loss in these cell types. Patch clamping is necessary to study the responses of specific BC types at single-cell resolution as previously established by Ichinose and Hellmer (Ichinose and Hellmer, 2016). How results from *in vitro* patch clamp studies inform results from *in vivo* ERG responses remains to be determined. Finally, due to the extremely large size of FAT3, we were not able to carry out the biochemical analysis needed to determine precisely how FAT3 affects PTPσ, GRIK1, and other uncharacterized candidate effectors (Avilés et al., 2022). Our discovery that CBC activity is compromised in *Fat3* mutants sets the stage for more detailed analysis in the future.

## Supplementary Material

Data S1 shows source data underlying all figures is provided in MS Excel format.

SourceData F6 is the source file for Fig. 6.

## Data Availability

The source data for graphs are provided with this paper and its online supplemental material (Data S1). The original images used to generate these data are available from the corresponding author upon request. The authors are happy to provide any materials including plasmids upon reasonable request.
