# Supplementary material for: ERG responses to high-frequency flickers require FAT3 signaling in mouse retinal bipolar cells
Source: J Gen Physiol. 2025 Feb 4;157(2):e202413642. doi: 10.1085/jgp.202413642 (PMC11793021; doi:10.1085/jgp.202413642)

Source data Figure 6

Full membrane. 3min exposure.
The sections shown in the paper are shown in yellow boxes

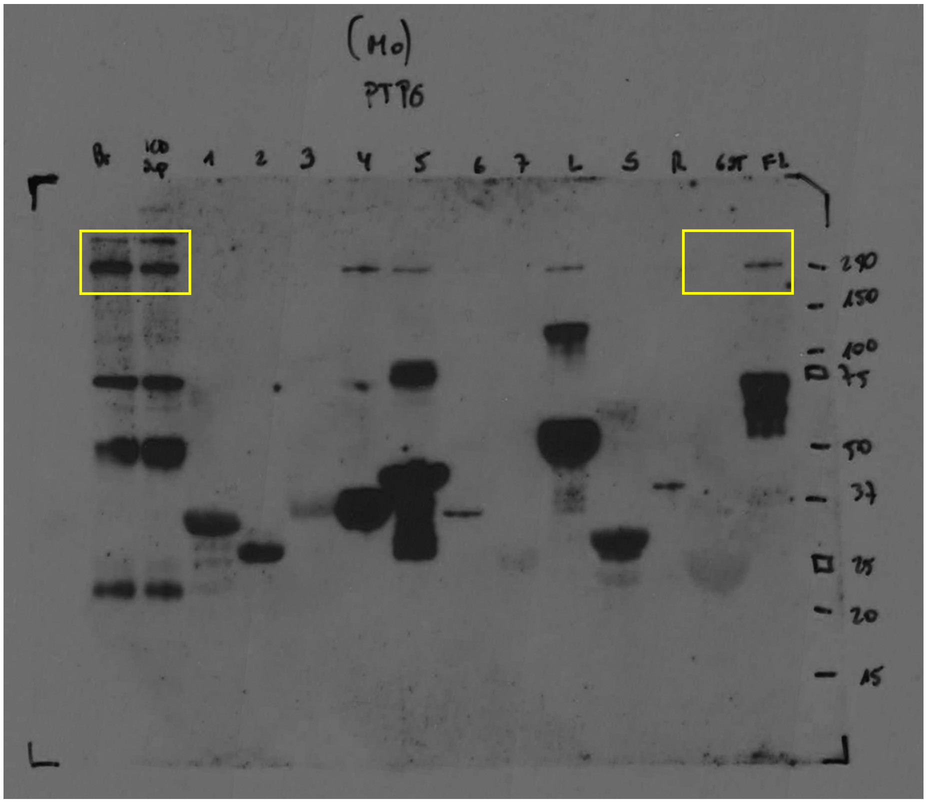

Supplement: SourceData F6 — is the source file for Fig. 6. [file jgp_202413642_sourcedataf6.pdf]
